# Statistically Classified Marine Epifaunal Bioregions on the Southern Benguela Margin, South Africa

**DOI:** 10.1002/ece3.72549

**Published:** 2025-12-09

**Authors:** Donia Wozniak, Lara Atkinson, Natasha Karenyi

**Affiliations:** ^1^ Department of Biological Sciences University of Cape Town Cape Town South Africa; ^2^ Department of Biological Sciences, Centre for Statistics in Ecology, Environment and Conservation (SEEC) University of Cape Town Cape Town South Africa; ^3^ South African Environmental Observation Network, Egagasini Offshore Node Cape Town South Africa; ^4^ Department of Biological Sciences, Marine and Antarctic Research Centre for Innovation and Sustainability (MARIS) University of Cape Town Cape Town South Africa

**Keywords:** assemblages, bioregionalization, bioregions, data‐driven, ecosystem classification and mapping, environmental drivers, marine epifauna, regions of common profile, South Africa, Southern Benguela

## Abstract

Marine bioregional maps often underpin effective management and conservation planning. Bioregional maps determined by statistical models can predict the most probable bioregion at unsampled locations and have many advantages over expert‐derived approaches, such as including measures of uncertainty. Here, we aim to statistically determine and map marine epifaunal bioregions on the Southern Benguela margin of South Africa, including their associated spatial uncertainty, utilising survey collected abundance data (70‐800 m). Regions of Common Profile (RCP) models were implemented using the ‘ecomix’ R package to model and map bioregions of epifaunal assemblages and their environment. Epifaunal abundance data from 46 species collected across 325 sites by research trawl surveys between 2017 and 2020, were used to inform bioregions. Environmental predictors were bottom dissolved oxygen and temperature measured in situ and slope derived from bathymetry. Five bioregions were identified. Two well‐defined depth breaks at ~150 m and ~400–500 m separated the mid shelf from the outer shelf and the outer shelf from the upper slope, respectively. The mid shelf and upper slope bioregions were associated with the highest estimated certainty, responding strongly to low oxygen concentration and low temperature, respectively. Outer shelf bioregions had the highest species richness, were dominated by the hermit crab *Sympagurus dimorphus* and were predicted with greater spatial uncertainty. This was the first study to statistically classify marine epifaunal bioregions using abundance data for the Southern Benguela margin of South Africa and furthers understanding of offshore epifaunal distributions and their potential drivers. Methods applied here produced practical outputs for spatial planning and conservation management by identifying five distinct epifaunal bioregions and associated environmental gradients. This study provides a foundation for conducting statistical approaches to marine ecosystem classification and mapping in South Africa.

## Graphical abstract

Marine bioregional maps underpin management and conservation planning. Data‐driven maps based on robust statistical analyses that include uncertainty are new to the marine realm as more data becomes available. The use of Regions of Common Profile models on epifauna data from the Southern Benguela margin distinguished five bioregions related to low oxygen and low temperature. The outputs of this study contribute to spatial planning and conservation management.

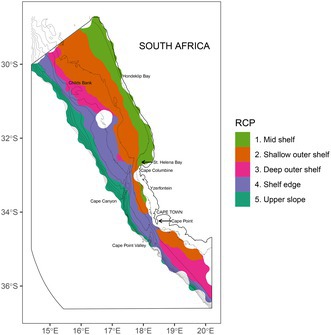

## 1 Introduction

Marine bioregional maps underpin a suite of management applications by providing ecologically relevant units to inform conservation planning (Harris, Holness, Kirkman, et al. 2022; McQuaid et al. 2023), identify ecologically and biologically significant areas (Gregr et al. 2012; Harris, Holness, Finke, et al. 2022), assess the level of ecosystem protection (Clark et al. 2011; Howell 2010; Kirkman et al. 2021; McQuaid et al. 2020; Ross and Howell 2013) and evaluate ecosystem threat statuses (Kirkman et al. 2019; Pitcher et al. 2018; Sink, van der Bank, et al. 2019). With several rising threats to marine ecosystems, there is an increasing need for responsible and effective management and conservation of marine resources and ecosystems (Watson et al. 2020). Here, bioregions are defined as regions in space explicitly determined by biological and environmental data, which are relatively more homogenous in species assemblage structure and environmental characteristics than neighbouring regions, at a particular scale of interest (Woolley et al. 2019).

Sampling the vastness of the marine environment is impractical due to high associated costs, technological challenges, and difficulty accessing certain areas (Diaz et al. 2004; Jamieson et al. 2013). This is often exacerbated in countries where the capacity for biological data collection may be under‐resourced (Sink et al. 2023). As a result, classifications and maps have often been informed by environmental data only, as surrogates for biological assemblages (e.g., Lombard et al. 2004; McQuaid et al. 2023; Roff et al. 2003; Spalding et al. 2007; UNESCO 2009). However, reliance on environmental surrogates alone can misrepresent biodiversity patterns, since abiotic variables do not always align with species distributions, may oversimplify fine‐scale ecological variation, and lack validation without biological data. Data‐driven or statistically determined bioregional maps improve estimation and assessment of biodiversity in data deficient areas, providing clearer inferences into species‐environment relationships when *explicitly defined by biological data* (Jetz et al. 2019; Przeslawski et al. 2011; Wang et al. 2021; Woolley et al. 2019).

Data‐driven approaches produce statistical groupings directly informed by georeferenced observations, offer greater reproducibility and are easier to automate than expert‐derived classifications and maps (Jetz et al. 2019; Rowden et al. 2018; Woolley et al. 2019). Numerical approaches which can quantify uncertainty in predictions, validate models and predict bioregions across spatial and temporal scales have particularly useful management applications (Woolley et al. 2019). While occurrence data (presence‐only or presence‐absence) are generally used to inform data‐driven bioregionalizations, since they are more easily modelled and accessible from open‐source databases, abundance data (counts, cover or biomass) improve knowledge of where species are likely to thrive and be most vulnerable by providing a continuous measure of species' contribution to community structure (Reiss et al. 2014; Stephenson et al. 2021). Compared to presence‐only data, abundance data collected via systematically designed surveys are less spatially biased and provide more robust information about species absences and environmental conditions (Austin 1998).

Numerous approaches to bioregionalization exist, and the purpose of mapped outputs should define what data to use or approach to implement (Costello 2009; Strong et al. 2019). Three broad approaches to data‐driven bioregionalization have been recognised, based on the order that classification and prediction steps occur (Ferrier and Guisan 2006). The ‘group first, then predict’ approach initially defines biological groupings, often using a method of ordination such as hierarchical cluster analysis (e.g., Rubidge et al. 2016), whereafter groups are related against environmental predictors using a species distribution modelling (SDMs) technique (Guisan and Zimmermann 2000). The ‘predict first, then group’ approach either uses multiple single SDMs or joint SDMs (JSDMs) which model multiple species simultaneously to relate species to their environment, after which clustering is performed to identify similar groups (e.g., O'Hara et al. 2011; Stephenson et al. 2021). Alternatively, classification and prediction may occur simultaneously within a single model for example, Species Archetype Models (SAMs, Dunstan et al. 2011; Hui et al. 2013), Regions of Common Profiles (RCPs; Foster et al. 2013) and Bayesian model‐based cluster analysis (ter Braak et al. 2003). Simultaneous approaches to bioregionalization allow model diagnostics to be performed and provide reliable estimates of uncertainty (Woolley et al. 2019).

Recent calls have been made for data‐driven approaches to assist with classification and mapping nationally in South Africa (Sink et al. 2023). Many ecosystems and their drivers are under studied and there are many challenges associated with accessing data that are at the desired spatial and temporal resolution (Sink et al. 2023). Abundance data collected annually by government‐led research trawl surveys have good potential for informing data‐driven bioregional maps due to their good spatial coverage across the western and southern continental margins of South Africa, compared to other sampling methods across the shelf for example, towed cameras (Wozniak 2023) and grabs (Karenyi et al. 2016). While these surveys are designed to target commercially important fish species, epifauna incidentally caught have been recorded since 2011. Here, epifauna are defined as invertebrates living on or just above the surface of the seabed or attached to submerged substrata, typically of macrobenthic size (approximately > 2 cm), large enough to be individually counted in ecological surveys (Rex 1981).

Epifaunal distributions are the focus of this study because their spatial patterns integrate ecological processes such as species interactions, environmental gradients and habitat heterogeneity, making them useful surrogates for benthic biodiversity in bioregional mapping (Bowden 2011; Buhl‐Mortensen et al. 2020; Howell 2010; Limongi et al. 2023; McArthur et al. 2009, 2010; Sink, Harris, et al. 2019; Stephenson et al. 2022). Epifauna typically have limited dispersal and strong associations with specific sediment types, depth ranges and hydrodynamic conditions (Atkinson, Field, and Hutchings 2011; Chen et al. 2021; de Juan et al. 2007; Jørgensen et al. 2011), so their community composition and turnover can highlight boundaries between ecologically distinct regions. These distributional patterns can thus be useful for delineating bioregions and identifying spatially structured benthic assemblages (Buhl‐Mortensen et al. 2010; Rex 1981).

This is the first study to use a data‐driven approach to predict and map epifaunal bioregions on the Southern Benguela margin of South Africa using abundance data collected by systematic surveys. This study aims to improve understanding of epifaunal patterns and drivers on the Southern Benguela margin of South Africa for the purposes of ecosystem classification and mapping by (i) applying a data‐driven approach to bioregionalization to define and describe epifaunal bioregions based on their species profiles and environmental responses; and by (ii) assessing uncertainty in spatial estimates.

## 2 Methods

### 2.1 Study Region

The Southern Benguela Shelf ecoregion lies on the western continental shelf of South Africa (Sink, Harris, et al. 2019). The cool, equatorward flowing Benguela Current characterises the western margin of South Africa (Shannon 1985). Dynamic wind‐driven upwelling predominantly occurs over the shelf in the austral summer and spring, enriching the top layers with nutrients (Shannon and Nelson 1996). High productivity supports large species biomasses and numerous important commercial fisheries (DEFF 2020; Payne et al. 1989). Commercial trawling efforts, targeting mostly hakes (*Merluccius* spp.), are concentrated on the shelf edge (Sink, Wilkinson, et al. 2012). Persistent Low Oxygen Waters (LOWs) resulting from decaying organic matter associated with high productivity are regular but variable features in the Namaqua subregion (north of Cape Columbine) and are most concentrated at depths shallower than 150 m (Jarre et al. 2015). The shelf is wide and gently sloping on the western margin, extending 240 km off the Orange River Mouth at the Namibian border, and is 50 km at its narrowest off Cape Point (de Wet and Compton 2021). The shelf edge break is one of the deepest globally, occurring between 200 and 600 m (de Wet and Compton 2021).

### 2.2 Data Collection

#### 2.2.1 Biological Data

**FIGURE 1:**
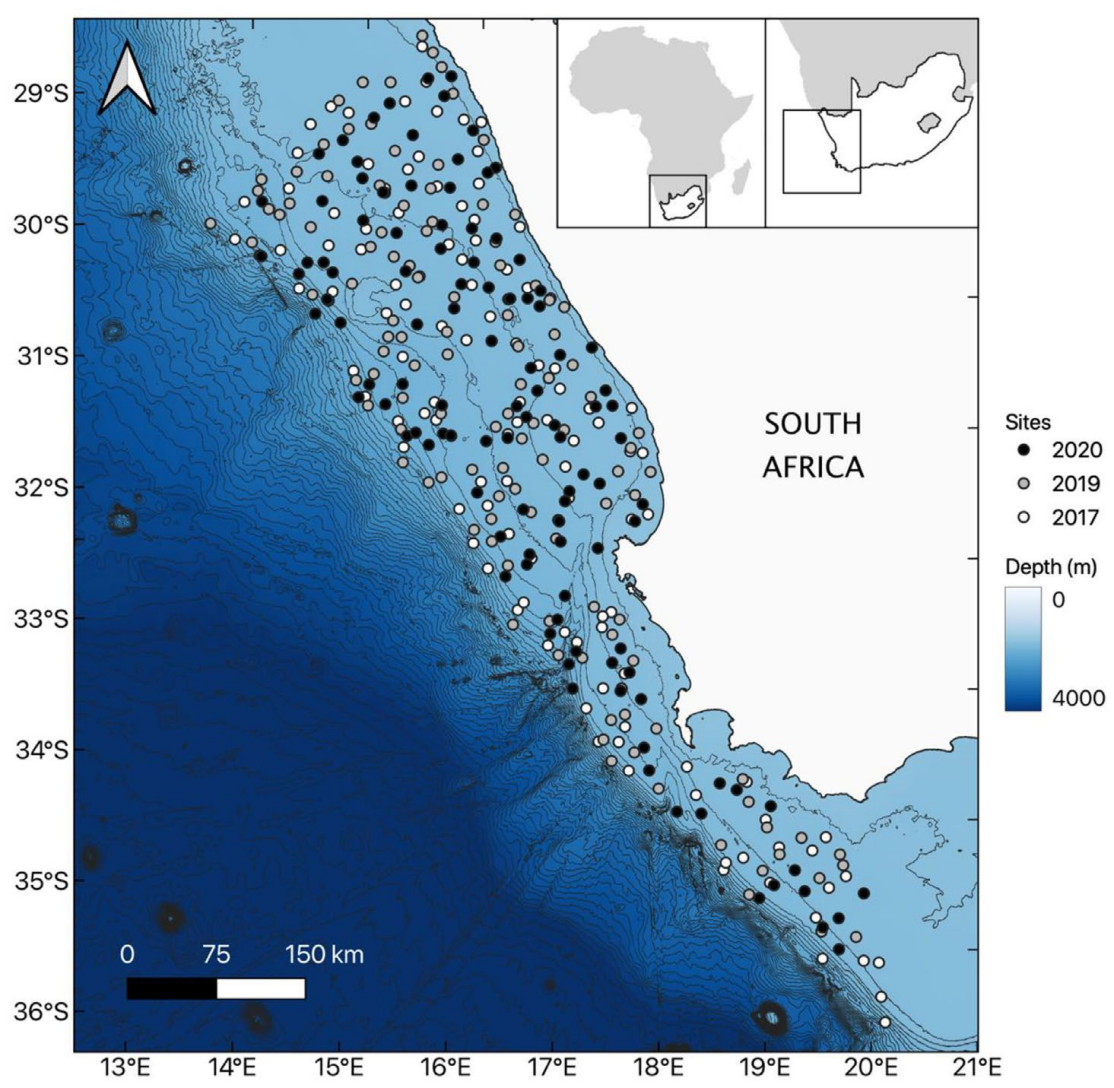
Study region located on the Southern Benguela shelf of South Africa showing bathymetric contours (100 m intervals). Map inserts show the location of the study area on the western margin of South Africa. Trawl sites are coloured by survey (year). The location of trawl sites is based on the start position of the 30‐min trawl tow.

Epifaunal data collected through a collaboration between the Department of Forestry, Fisheries and Environment (DFFE) and the South African Environmental Observation Network (SAEON) during annual demersal research trawl surveys across the western margin of South Africa (up until 20° E) was used to model bioregions (Figure 1). Trawl sites used here were collected from ~70 to 800 m over an area of ~118,000 km^2^. Data were obtained from 3 years of surveys (2017, 2019 and 2020) conducted on board the *FRS Africana* during the austral summer (Figure 1).

Epifaunal abundance (counts) was collected using a pseudo‐random depth stratified survey design, with the number of sites in each depth and longitude stratum being directly proportional to the area of each stratum (Atkinson, Leslie, et al. 2011; Badenhorst and Smale 1991; Yemane et al. 2008). Trawling occurred during daylight hours only (from 30 min after sunrise to 30 min before sunset) and trawls were towed at a constant speed of 3.5 knots for 30 min (bottom time). The trawl gear consisted of a four‐panel 180 ft (55 m) German otter trawl net, with 9 m sweeps and 1.5 t Morgere multipurpose otter boards (Atkinson, Leslie, et al. 2011; Yemane et al. 2008). The 75 mm mesh cod‐end was lined with a sleeve of pilchard netting (35 mm mesh; Yemane et al. 2008) for retaining large and small fish and consequently many of the invertebrates. The trawl's door spread was approximately 60–75 m, with a vertical mouth opening of 3–4 m and a horizontal mouth opening of 20–29 m (Atkinson, Leslie, et al. 2011). All benthic invertebrates retained in the trawl net were recorded, sorted to the lowest possible taxon, counted and weighed. Species identifications were cross‐referenced in the World Register of Marine Species (WoRMS Editorial Board 2023).

Prior to data cleaning, the raw biological dataset consisted of a total of 535,691 individuals from 274 taxa recorded at 358 sites during the 3 years of trawl surveys. Taxa which were not reliably identified to species level or verified as distinct species were removed. These included unknown species, pelagic species and unverified abundance records (31%). Rare species do not necessarily add important information for bioregionalization and were therefore excluded from modelling to remove data noise (Foster et al. 2017; Hui et al. 2013). Due to the large number of rare species in the dataset (20% of species occurred at one site only), species were considered rare if they occurred at fewer than 6% of sites. This was based on previously applied thresholds for rarity at 1%–5% of the dataset (Foster et al. 2017; Hill et al. 2017; Lyons et al. 2017; Woolley et al. 2019). Sites with < 3 species recorded were excluded as such low‐diversity records may not adequately represent a community profile and are considered to contribute little information for subsequent RCP modelling (Foster et al. 2013). After filtering, a total of 303,289 individuals from 46 epifaunal taxa (see Table S1.1) recorded at 325 sites were used to inform the final model (24% of valid species records).

#### 2.2.2 Environmental Data

Selecting appropriate predictor variables that are both ecologically meaningful and explain a significant proportion of the biological variation is important when developing models (Austin 2002). Freely available, comprehensive and validated environmental layers for key abiotic seabed variables of the South African EEZ are not always available at finer resolutions, and may be interpolated from limited samples or satellite data for example, Bio‐ORACLE (Assis et al. 2017; Tyberghein et al. 2012) which can lead to spurious results. Only reliable environmental layers derived from oceanographic measurements in situ (depth, temperature, pressure, salinity and oxygen) and slope derived from the best available bathymetry layer were therefore considered as predictors for this study (Table 1).

**TABLE 1:** Environmental predictor variables (continuous) considered for modelling epifaunal bioregions. Range values based on in situ measurements at the seafloor.

Variable	Unit	Range	Data source	References
Temperature*	°C	3.8–10.4	Fisheries independent survey collected via in situ CTD	DFFE (2017, 2019, 2020) (Conductivity, Temperature and Depth profiles collected during South African Department of Forestry, Fisheries and Environment West Coast demersal research surveys. Unpublished data.)
Pressure	db	71.1–864.4	As above	As above
Salinity	PSU	34.2–35.0	As above	As above
Oxygen*	mL/L	0.1–5.1	As above	As above
Depth	m	70.6–856.7	As above	As above
Slope*	degrees	0.0–8.4	Derived from single‐beam bathymetry	de Wet and Compton (2021)

*Note:* Environmental covariates used in the final model are indicated by an asterisk (*).

Oceanographic variables used for modelling were obtained from the same years of surveys as the biological data and were collected in situ via an SBE 19plus V2 SeaCAT Profiler CTD (Sea‐Bird Scientific 2022) attached to the headline of the trawl net. Values assigned to each trawl represented the average value of each oceanographic variable along the bottom profile of the trawl. The ‘Slope’ tool from the ‘Spatial Analyst Toolbox’ in ArcGIS Pro version 2.9.0 (ESRI) was used to derive slope values from bathymetry compiled from digital single‐beam echo sounding data (de Wet and Compton 2021). Slope values were assigned to each trawl site using the location of the trawl start point. Highly correlated covariates (Pearson correlation coefficient of *r* > 0.7) were excluded from the analysis since strong correlations between predictor variables can confound modelling (see Figure S2.1). Among the highly correlated variables (depth, pressure, salinity and temperature), temperature was retained as the most proximal variable to the distribution of benthic epifauna, in order to improve model robustness and ecological relevance (Austin 2002; Kenchington et al. 2019). After excluding highly correlated variables, the environmental covariates included as predictors for modelling epifaunal bioregions were bottom temperature (°C), bottom dissolved oxygen (mL/L) and slope (°).

Gridded raster layers of environmental variables were produced at a resolution of 0.004° to predict bioregions spatially (see Figure S2.2). Universal kriging was used to interpolate the site‐wide oceanographic variables using the ‘gstat’ R package (Pebesma 2004) in RStudio v. 4.10 (RStudio Team 2021) and raster layers were combined into a multidimensional (stacked) raster layer for spatial prediction using the ‘raster’ R package (Hijmans et al. 2022).

### 2.3 Data Analysis

The Regions of Common Profile (RCP) modelling framework (Foster et al. 2013, 2017) was applied to statistically determine bioregions and estimate their associated species profiles and environmental responses. RCP models can utilise both presence‐absence and abundance data to identify similar sites based on their environmental response profiles and species catch profiles (i.e., mean expectation or abundance of all species belonging to an RCP) by explicitly incorporating information about species locations (Foster et al. 2013, 2017). Sites are classified as belonging to one of a finite number of RCP groups and all sites within the same RCP group are assumed to share a constant species profile. RCP models follow a simultaneous approach to classification and prediction, allowing uncertainty to be quantified and mapped (Woolley et al. 2019).

#### 2.3.1 Model Fitting and Group Selection

The ‘ecomix’ R package (Woolley 2022) was used to fit RCP models and analyses were adapted from Woolley (2021). To fit the models, continuous environmental covariates (temperature, oxygen and slope) were centred and standardised into 2nd degree orthogonal polynomials by adding a quadratic term for each variable. Epifaunal abundances were modelled with a negative binomial error distribution. Model parameters were estimated using penalised maximum likelihood (Foster et al. 2013). For the likelihood to optimise and avoid finding local maxima, 100 multiple fits were performed. A control was added to help smooth the log‐likelihood by setting several mild penalties on the estimated parameters (Foster et al. 2017).

To determine the optimum number of RCP groups, Bayesian information criterion (BIC) was used to select the best model. A likely range of RCP groups was set prior to modelling, 2–8 groups, and the optimum number of groups was determined based on the model that minimised BIC (Foster et al. 2013). The maximum number of groups was limited to eight to avoid overfitting, particularly given the limited number of environmental predictors (*n* = 3) which reduces the scope for reliably distinguishing a larger number of groups. To avoid selecting a model with RCP groups that have low site affinity, site memberships of RCP groups (sum of posterior probabilities) were inspected for all models to ensure no groups were poorly represented and that all sites had indeed been classified into an RCP.

#### 2.3.2 Estimating Bioregions and Uncertainty

Bayesian bootstrapping (Rubin 1981) was used to estimate uncertainty in parameter estimates and predictions (Foster et al. 2017). Confidence intervals around the point predictions were taken as a symmetric 95% interval from a bootstrap distribution of 100 iterations. To predict expected abundances (number of individuals) of species at an average site for each RCP (±confidence intervals), species catch profiles were calculated directly from model coefficients.

Probabilistic maps of the spatial distribution of each of the RCPs (±confidence intervals around point estimates) were predicted in environmental space across the study area. A hard classification (non‐probabilistic) was produced by fixing an RCP type in space, based on the most likely RCP at each point in space. Species catch profiles were estimated for each bioregion, represented by the mean ± standard deviation (SD) of species abundances for an average site. Plotted species catch profiles were represented on the log scale (by defining a ‘link’ response) for easier visual inspection of the group contents for each RCP. Environmental responses of each RCP were quantified from model coefficients and assessed with partial plots. For partial response plots, the value of each environmental covariate was kept at the mean value, except for the variable of interest. Code and additional functions used to plot the partial responses were adapted from Hill et al. (2020).

#### 2.3.3 Model Diagnostics

The best model was assessed through various diagnostic tests. These included random quantile residuals (RQR; Dunn and Smyth 1996) adapted for mixture models (Dunstan et al. 2013) to assess variation and Quantile‐Quantile (QQ) plots to check for normality of residuals (Foster et al. 2017). Since RCP groups are latent (unobserved), traditional validation of how well the RCP groups represent the data is difficult to perform. However, the robustness and stability of RCP groups and log‐likelihoods can be assessed by removing subsets of the data of increasing size. The Cook's distance metric (Cook 1979) was used to assess the stability of RCP groups (Foster et al. 2017).

## 3 Results

Five RCP bioregions based on bottom temperature, bottom dissolved oxygen concentration and slope were defined by selecting the model which minimised BIC (see Figure S3.3). Model diagnostics indicated an acceptable performance as variation was reasonably well captured by the model and the predictive log‐likelihood remained adequately stable when subsets of the data were removed (see Figures S3.4 and S3.5). Sites were reasonably distributed within each bioregion as indicated by the sum of posterior probabilities (Table 2).

**TABLE 2:** Summary of characteristics of the five epifaunal bioregions predicted across the southern Benguela shelf of South Africa. Predicted number of n sites are based on the sum of the posterior probabilities over sites. Depth across shelf values are based on the 5th and 95th percentiles of depths within each bioregion to reduce the influence of outliers. Environmental response denotes the predictors that bioregion had a strong response to (> 0.5 probability of occurring at a given range of that predictor). Environmental characteristics shows the most likely temperature (°C), oxygen (mL/L) and slope (°) values with the associated probability in brackets. Average species richness and total abundance metrics were derived from species catch profiles, with values representing the expected mean abundance profile across species. Indicator species are defined as having a mean abundance of > 1 individual per site in only one bioregion across predicted species profiles.

RCP	Shelf position	*n* sites	Area (km^2^)	Depth across shelf (m)	Environmental response	Environmental characteristics	Average species richness	Average total abundance	Most abundant species (mean abundance)	Indicator species
RCP1	Mid shelf	53	19,698	32–160	Oxygen	10.43°C (0.45) 0.11 mL/L (0.81) 0.00° (0.01)	9	2364.42	*Pasiphaea* sp.1 (1906.28) *Pterygosquilla capensis* (246.23) *Cavernularia* spp. (183.81)	*Athlete lutosa* *Cavernularia* spp. *Pasiphaea* sp. 1
RCP2	Shallow outer shelf	98	39,800	138–253	Temperature	9.09°C–9.56°C (0.56) 2.14–2.96 mL/L (0.22) 0.00° (0.37)	12	531.86	*Sympagurus dimorphus* (363.27) *Pterygosquilla capensis* (110.62) *Spatangus capensis* (14.77)	*Mediaster bairdi capensis*
RCP3	Deep outer shelf	66	16,023	163–326	Oxygen	8.88°C–9.36°C (0.34) 5.14 mL/L (0.62) 0.00° (0.25)	26	576.59	*Sympagurus dimorphus* (466.32) *Spatangus capensis* (27.47) *Psilaster acuminatus* (18.37)	*Cosmasterias felipes* *Echinus gilchristi* *Ophiothrix aristulata* *Pteraster capensis* Velutinid
RCP4	Shelf edge	51	28,519	219–491	Temperature Oxygen Slope	6.79°C–7.2°C (0.89) 2.75–2.85 mL/L (0.66) 7.51°–7.59° (0.98)	24	1043.29	*Sympagurus dimorphus* (695.54) *Crossaster penicillatus* (83.81) *Parapagurus bouvieri* (79.03)	
RCP5	Upper slope	57	15,538	426–1000	Temperature	4.16°C–4.50°C (0.95) 0.77–1.13 mL/L (0.22) 2.99°–3.50° (0.15)	15	640.79	*Crossaster penicillatus* (413.24) *Sergia* spp. (81.13) *Chaceon chuni* (48.30)	*Chaceon chuni* *Hyalinoecia tubicola* *Plesionika martia* *Sergia* spp.

### 3.1 Estimated RCP Distributions

**FIGURE 2:**
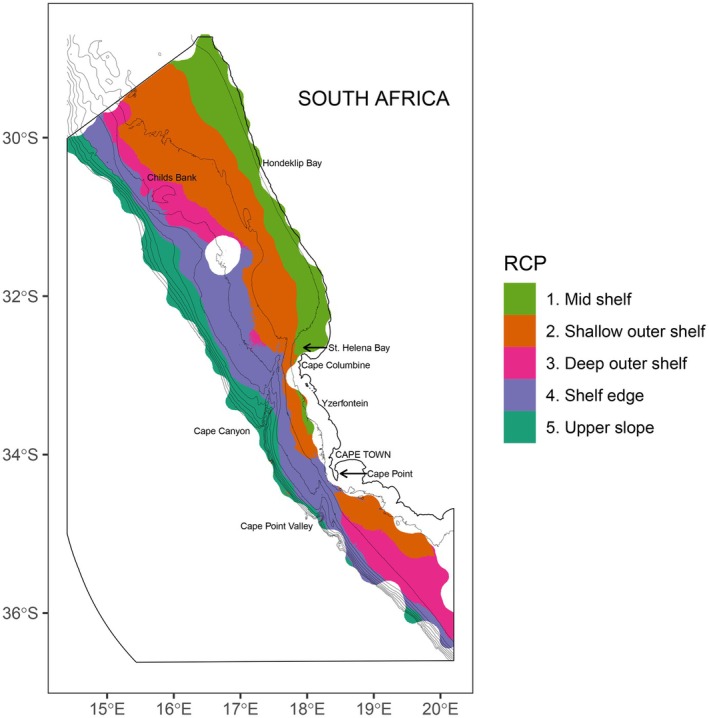
Predicted spatial distributions of RCP groups to produce a hard (non‐probabilistic) classification across the environmental space of the study area. Bathymetry contours are shown in 100 m intervals from 100 to 1000 m depth. Bioregions (RCP 1–5) are separated by colour and based on a model using 46 epifaunal species from 325 sites and three covariates. White gaps indicate hard ground and inshore areas that are not trawled during surveys.

The five RCPs followed a depth gradient across the shelf (Figure 2). The shallowest bioregion, RCP 1 (light green), occurred across the mid shelf (19,698 km^2^) and was most likely to occur between depths of 32 and 160 m (Table 2). This was followed by RCP 2 (orange) which occurred across the shallow outer shelf (138–253 m) and covered the greatest area (39,800 km^2^). RCP 3 (pink) was distributed across the deep outer shelf (16,023 km^2^) from 163 to 326 m and RCP 4 (purple) across the shelf edge (28,519 km^2^) from 219 to 491 m. The deepest bioregion, RCP 5 (dark green), occurred across the upper slope at depths greater than 426 m and covered the smallest area of 15,538 km^2^ (Table 2, Figure 2).

**FIGURE 3:**
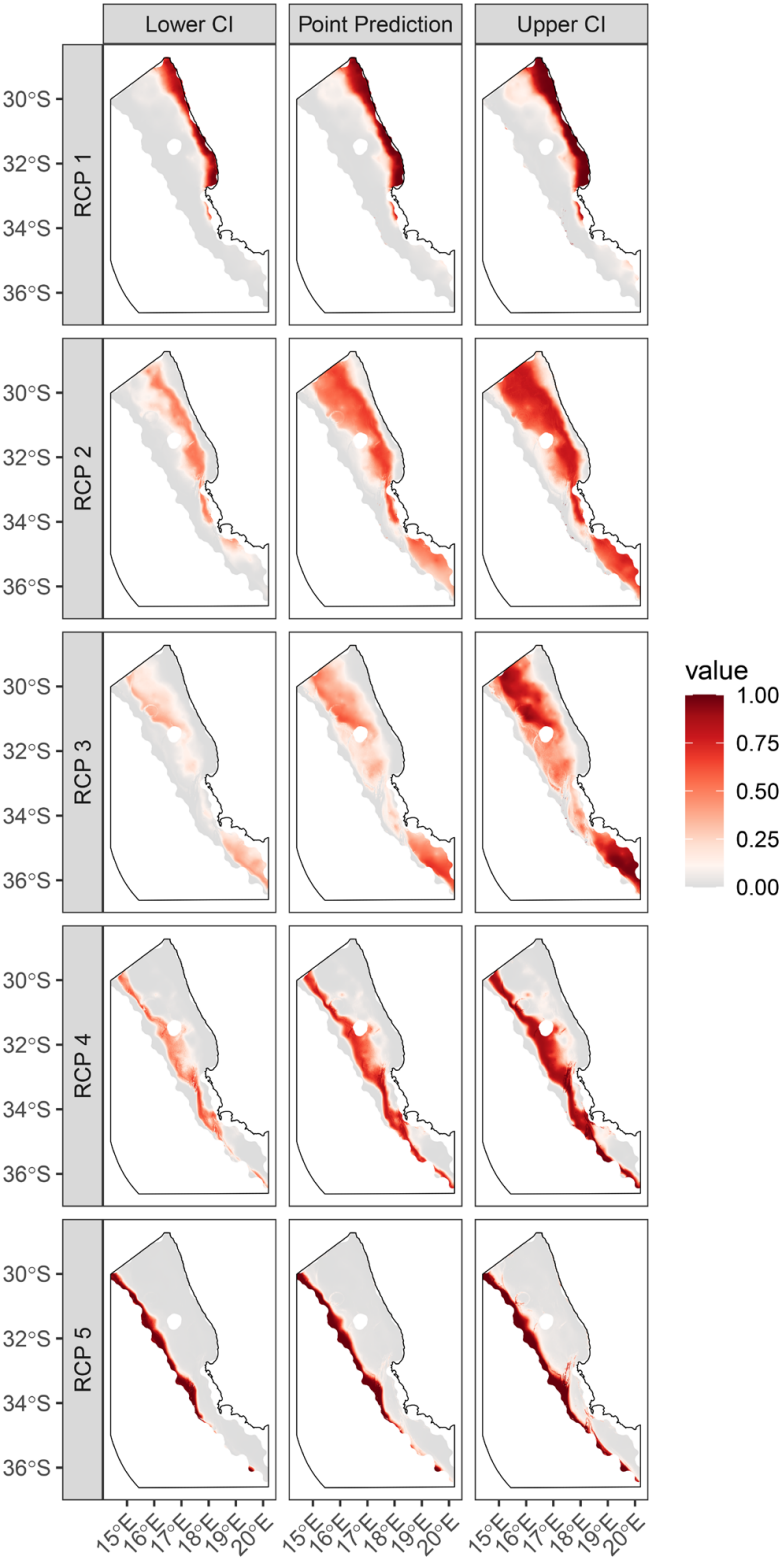
Predicted spatial distributions (±SE) of RCP bioregions across the environmental space of the study area. RCPs (1–5) are separated by rows. Middle panels represent the spatial distribution of the point predictions (mean) for each RCP, while uncertainty is represented by bootstrapped 95% confidence intervals (right and left panels). RCPs are based on a model using 46 epifaunal species from 325 sites and three covariates. RCP probabilities are shaded from low (grey) to high (dark red).

The upper slope (RCP 5) and mid shelf (RCP 1) bioregions were predicted across the shelf with a high degree of certainty, as indicated by probability values close to 1 (dark red) in both the upper (right) and lower (left) confidence interval (CI) panels (Figure 3). Outer shelf bioregions (RCP 2, 3 and 4) over intermediate depths (~150–500 m) were associated with lower degrees of certainty (wider CIs) as indicated by light red colours in the lower CI panels and darker red colours in the upper CI panels. The deep outer shelf (RCP 3) overlapped substantially in space with the shallow outer shelf (RCP 2) and the shelf edge (RCP 4), and was predicted with the least amount of certainty, with lower probabilities (light red colours) of prediction. All bioregions were least certain around their boundaries, as seen by the lightest red colours occurring towards the edges of bioregions (Figure 3).

### 3.2 RCP Species Profiles and Environmental Responses

**FIGURE 4:**
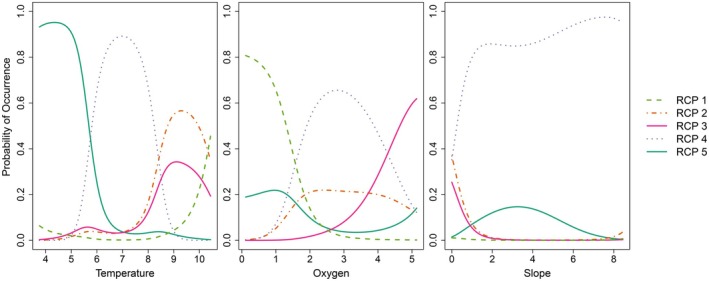
Partial effects plots for the effect of each of the continuous environmental variables: Temperature (°C), oxygen (mL/L) and slope (°), on the probability of occurrence of the five epifaunal RCP bioregions across the study area. RCPs are based on a model using 46 epifaunal species from 325 sites and three covariates. Covariates were held at their mean values to make the predictions.

All RCP bioregions responded strongly to at least one of the environmental predictors tested. Temperature varied across the shelf from 3.76°C to 10.44°C, dissolved oxygen from 0.11 to 5.14 mL/L and slope from 0.002° to 8.448° (Figure 4). The upper slope bioregion, RCP 5, had the strongest responses to temperature, with a high probability (0.95) of occurring in the coldest bottom temperatures of the study region (4.16°C–4.50°C, Table 2). The upper slope was more likely to be found in low oxygen waters than in oxygenated waters (0.77–1.13 mL/L), though only with a low probability of 0.22. The mid shelf bioregion (RCP 1) responded most strongly to oxygen, with a high probability (0.81) of occurring in low oxygenated waters (0.11 mL/L). The warmest temperature (10.43°C) was associated with the mid shelf. The mid shelf had the lowest response to slope, with a probability of < 0.02 of occurring at any of the slope values in the study area (Figure 4, Table 2).

Among the outer shelf bioregions, RCP 2 and 3 were predicted to occur across similar values of temperature (8.88°C–9.56°C) and slope (0.00°, Figure 4, Table 2). The deep outer shelf (RCP 3) had a greater response to oxygen compared to the shallow outer shelf (RCP 2), with a relatively high bottom oxygen concentration of 5.14 mL/L (0.62 probability) predicted for the deep outer shelf. The shelf edge (RCP 4) had a high probability (0.89) of occurring in bottom temperatures between 6.79°C and 7.20°C and a moderate probability (0.66) of occurring in waters with a bottom oxygen concentration between 2.75 and 2.85 mL/L. The shelf edge was the only bioregion with a strong response to slope, with a high probability (> 0.8) of occurring at all slope values greater than 1° and most likely to occur at slope values of 7.51°–7.59° (Figure 4, Table 2).

**FIGURE 5:**
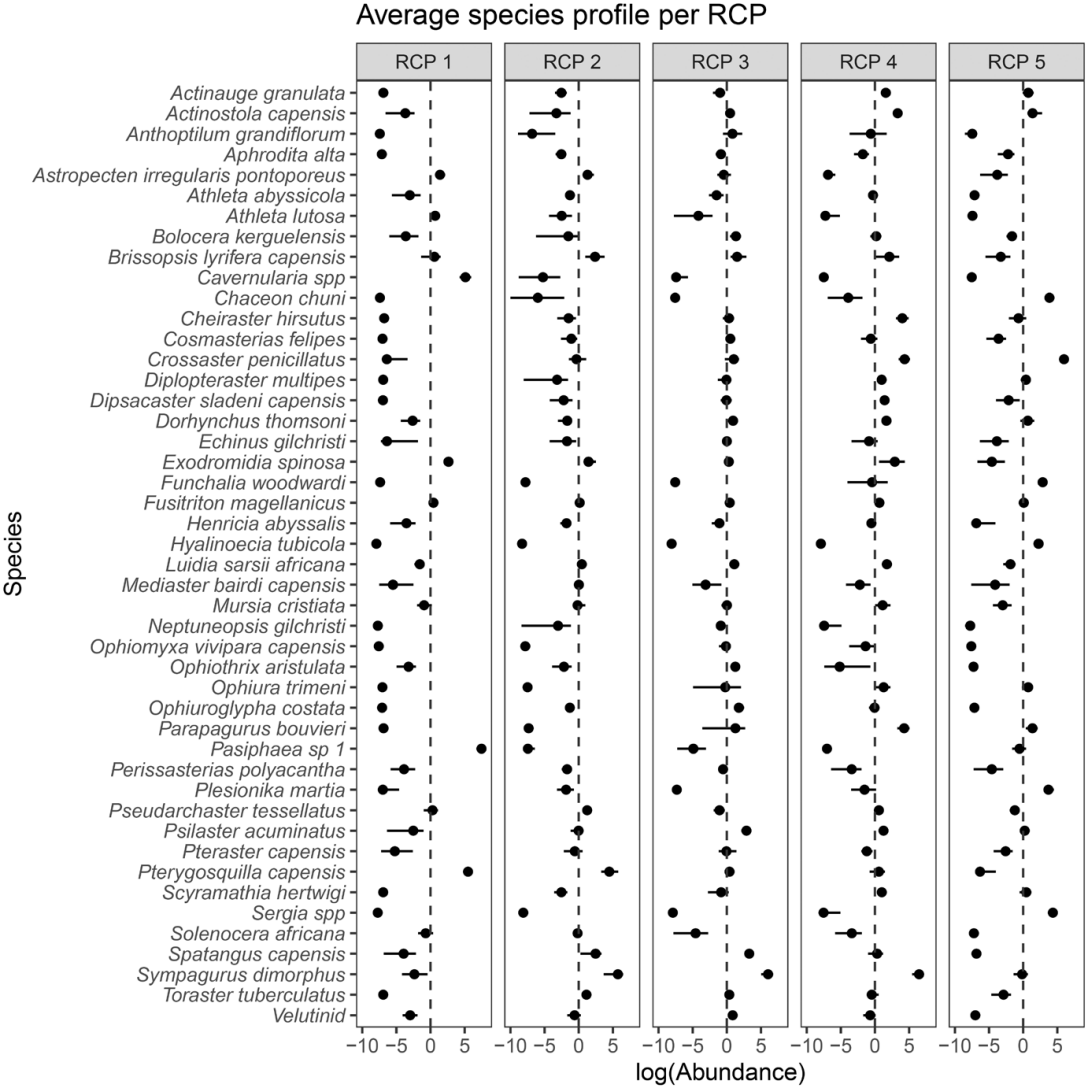
Average species catch profiles, the estimated mean abundances of species predicted at a site in each bioregion (RCP 1–5). Abundances are represented on the link scale (log abundance) ± lower and upper confidence intervals (CI) for easier visualisation. Predicted abundances are based on a model using 46 epifaunal species and three environmental predictor variables (temperature, oxygen, and slope) from 325 sites. Note that species associated with negative log(abundance) values indicate estimated abundances of less than 1 individual per site, but greater than 0 (see Table S4.2).

**FIGURE 6:**
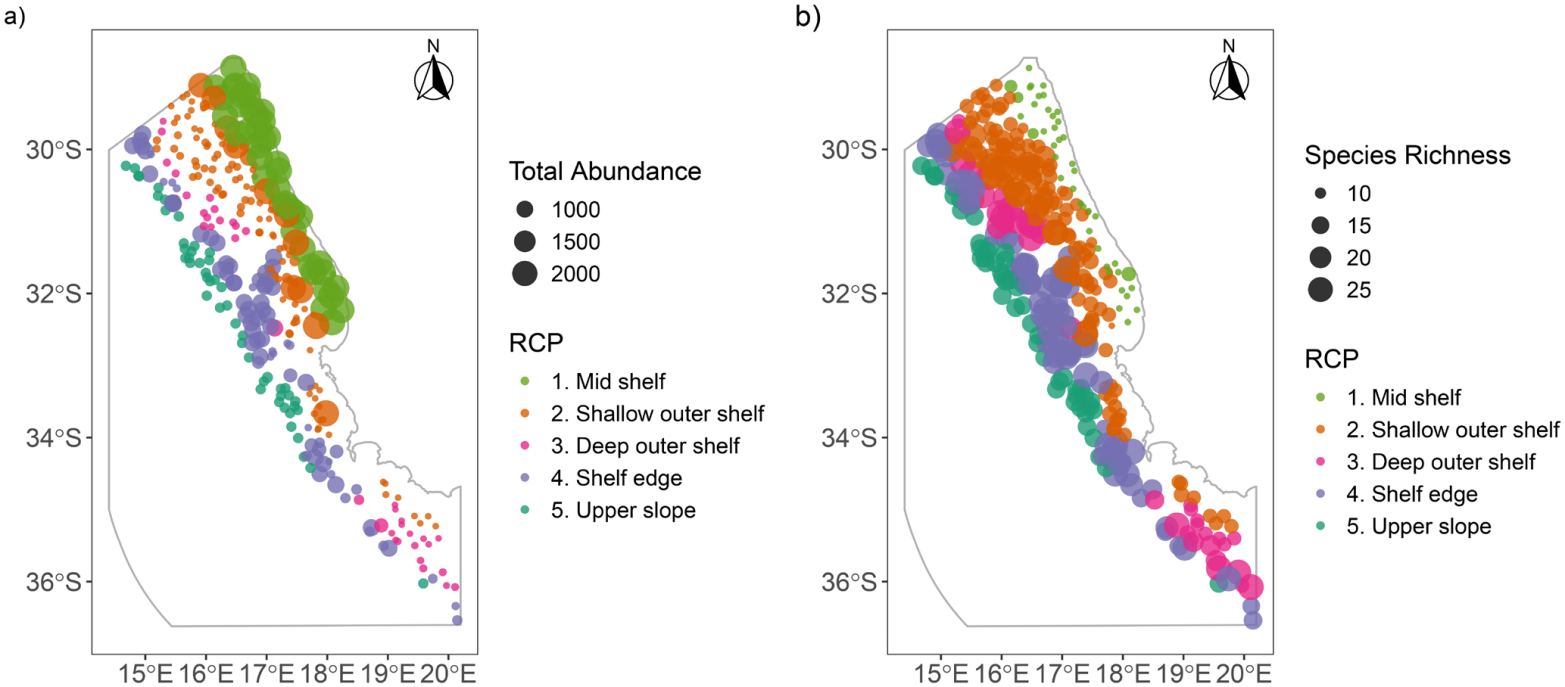
(a) Total predicted abundance per site (sum of total species abundance per site) and (b) total richness per site for each epifaunal bioregion. Predicted abundance per site was calculated as the sum of predicted abundances across all species using model outputs assigned to bioregions according to the hard RCP classification. A reasonable cut‐off of > 1 individual per site was assumed to infer richness at that site. Circle size represents abundance weight, with larger circles denoting a relatively greater abundance or richness at that size. See Figure S4.6 for individual species' contributions to total abundance.

All species were clearly associated with one or more bioregions, except for three species ( *Perissasterias polyacantha* , *Aphrodita alta*, *Neptuneopsis gilchristi*) which occurred at the lowest average abundances throughout the dataset (Figure 5, see Table S4.2 for values). Species with the highest predicted average abundances across the mid shelf (RCP 1) or the upper slope (RCP 5) tended to be indicative of those bioregions, occurring nowhere else (Table 2). These included the relatively abundant crab *Chaceon chuni* (48.30 ind.) and prawn species *Sergia* spp. (81.13 ind.) across the upper slope; and the sea pen *Cavernularia* spp. (183.81 ind.) and glass shrimp *Pasiphaea* sp. 1 (1906.28 ind.) across the mid shelf. The highest abundances across sites were found nearest the coast, across the mid shelf and the shallow outer shelf region adjacent to it and were largely driven by the highly abundant shrimp *Pasiphaea* sp. 1 (Figure 6a, see Figure S4.6 for individual species abundances). However, the mid shelf bioregion (RCP 1) was the least species‐rich, with 9 species predicted to occur with mean abundances greater than 1 (Figure 6b, Table 2). While species richness was lowest near the coast, it increased further offshore, peaking across the deep outer shelf (RCP 3) and shelf edge (RCP 4), with 26 and 24 species respectively predicted to occur with mean abundances greater than 1 (Figure 6b, Table 2).

The deep outer shelf (RCP 3) supported the highest number of indicator species (five), most of which were Echinoderms, whereas no indicator species were associated with RCP 4, the shelf edge (Table 2). Despite this, overall abundances were lowest across the deep outer shelf and adjacent regions of the shallow outer shelf (Figure 6a). The dimorphic hermit crab *Sympagurus dimorphus* emerged as the most abundant species on average in bioregions of intermediate depths, contributing substantially to the total abundance across the outer shelf (Figure 5, Figure S4.6). Estimated mean abundances of *S. dimorphus* were highest over the shelf edge (RCP 4: 695.54 ind.), with comparatively lower values in the shallow (RCP 2: 363.27 ind.) and deep outer shelf bioregions (RCP 3: 466.32 ind., Table 2). Both hermit crabs *S. dimorphus* and *Parapagurus bouvieri* were estimated to peak in abundance over the shelf edge; however, *S. dimorphus* was more abundant than *P. bouvieri* in shallower bioregions, while *P. bouvieri* was more abundant than *S. dimorphus* in the deeper upper slope bioregion (Figure 5).

## 4 Discussion

Determining species distributions and their drivers improves knowledge of biogeographic patterns and ecological processes which have implications for management (Hewitt et al. 2004; Williams et al. 2010). Statistical approaches to classification and mapping provide objective and practical frameworks for predicting bioregional maps for conservation management (Reiss et al. 2014; Woolley et al. 2019). This study identified marine epifaunal bioregions (areas with similar species profiles and environmental characteristics) on the South African Southern Benguela margin furthering knowledge of benthic distribution patterns and drivers to facilitate conservation mapping and planning.

Five marine bioregions based on trawled epifaunal abundance were defined by applying an RCP framework (Foster et al. 2013, 2017). Boundaries between bioregions occurred at bathymetric breaks typically identified across continental shelves, such as the shelf‐slope transition zone which usually occurs at 300–500 m globally (Carney 2005). In this study, epifaunal patterns showed a clear boundary between the upper slope and shelf edge at ~400–500 m, where the geomorphology of the shelf breaks on the western margin (de Wet and Compton 2021; Emery et al. 1992). This depth zone also coincides with marked faunal changes previously described for cephalopod (Roeleveld et al. 1992), demersal fish (Atkinson, Leslie, et al. 2011; Roel 1987), macrofauna (Karenyi 2014) and epifauna (Lange and Griffiths 2014; Shah 2018).

Sudden changes in the local environment (e.g., temperature, pressure) usually occur at transition zones. Here, low temperatures (4.16°C–4.50°C) were associated most closely with the upper slope bioregion (RCP 5). Changes in temperature often correspond to observed zonation patterns of epifaunal communities and low temperatures have previously been linked to upper slope communities both locally (Roeleveld et al. 1992; Smale et al. 1993) and on the western margin of Australia (Williams et al. 2010). In this study, species predicted to occur with high abundances across the upper slope, such as the *Crossaster* spp. starfish, are known to occur at low temperatures associated with the shelf‐slope boundary (Ringvold and Moum 2020). However, different taxa appear to be associated with continental slopes compared to the shelves, even in the absence of temperature variation (Carney 2005). Fauna respond to a wide variety of factors which covary with depth (Rex 1981) and in deeper environments decreasing temperatures and increasing hydrostatic pressures appear to contribute to observed faunal depth strata (Brown and Thatje 2014).

Though environmental drivers of benthic diversity patterns have been well documented (e.g., Carney 2005; Levin et al. 2001; Rex and Etter 2010), studies on the specific dynamics influencing offshore epifaunal distributions along the Southern Benguela margin of South Africa remain limited. Previous trawl‐based studies have delineated six epifaunal communities on the west coast shelf (Lange and Griffiths 2014) and 14 biotopes in the same region (Shah 2018), with depth consistently emerging as a key factor structuring these assemblages. However, given the strong correlation of depth with many environmental variables, it was excluded from our models in favour of predictors more directly linked to epifaunal distributions (i.e., temperature and oxygen).

Sharp changes in temperature may also reflect a transition in water masses, as temperature is a property associated with different water masses. Water mass features often explain distributional patterns of epifauna globally (Buhl‐Mortensen et al. 2020; Limongi et al. 2023; Neumann et al. 2017; Snelder et al. 2007; Williams et al. 2010), as well as locally on the western continental slopes of South Africa where certain species of cephalopod have been associated with cold (3°C–4°C) Antarctic Intermediate Waters (AAIW) (Roeleveld et al. 1992). Intensified erosional currents are also associated with upper slopes in general (Levin et al. 2001). Such currents create disturbance, influence food supply (Levin et al. 2001) and may inhibit larval transport between shelf and slope species (Carney 2005). These oceanographic features likely contribute to the shelf‐slope boundary observed here for epifaunal communities.

A local depth break at ~150 m was also identified at the boundary between the mid shelf (RCP 1) and the shallow outer shelf (RCP 2), where changes in local communities have previously been identified for epifauna (Lange and Griffiths 2014; Shah 2018), macrofauna (Karenyi 2014) and fish and cephalopods (Smale et al. 1993). This mid shelf bioregion was spatially well aligned with the ‘Namaqua shelf’ described in previous ecosystem classifications for South Africa (Sink, Holness, et al. 2012; Sink, Harris, et al. 2019). This bioregion was characterised by low oxygen concentrations (< 0.3 mL/L) which feature regularly across the Namaqua shelf region due to decaying phytoplankton driven by seasonal upwelling (Jarre et al. 2015). The lowest species richness was estimated for the mid shelf, as low oxygen zones in general tend to be less diverse due to higher physical stress (Levin 2003). Those species that were predicted to occur at high abundances are likely more tolerant of low oxygen concentrations than other species, such as the mantis shrimp *P. capensis* (Abelló and Macpherson 1990).

The highest certainty in predictions was associated with the mid shelf (RCP 1) and the upper slope (RCP 5), where depth breaks at ~150 and 400–500 m were defined, respectively. Bioregions at intermediate depths of the outer shelf were predicted with higher degrees of uncertainty in spatial estimates and they were not as clearly delineated from one another along isobaths as the upper slope and mid shelf bioregions. Communities are not spatially restricted entities (Leaper et al. 2014) and species overlap was found between all bioregions; however, species profiles estimated for the upper slope and mid shelf had a high proportion of indicator species. Similarly, an assemblage associated with the continental slope off British Columbia was found to be more distinct than shallower shelf assemblages and had the highest degree of indicator species (Rubidge et al. 2016). In contrast, no indicator species were predicted to occur across the shelf edge, where a variety of deep and shallow species usually occupy the shelf–slope transition zone (Carney 2005).

Outer shelf bioregions (RCP 2, 3 and 4) were dominated by the same highly abundant species; the hermit crab, *Sympagurus dimorphus*, suggesting that environmental thresholds are less severe across the outer shelf than they are at 150 m and 400–500 m on the western margin. *S. dimorphus* has previously been identified as highly abundant across the outer shelf of South Africa's western margin from 200 to 500 m (Lange and Griffiths 2014; Wright et al. 2020) and is perhaps indicative of this outer shelf region. These findings suggest that strong environmental processes at 150 m and 400–500 m (such as the ones discussed above) on the Southern Benguela margin of South Africa influence epifaunal tolerances or inhibit their spatial movement across these boundaries in some way, leading to the observed patterns.

The higher uncertainty in spatial predictions for bioregions at intermediate depths could also suggest that important environmental predictors for the outer shelf were not captured in this study. Since both the deep outer shelf (RCP 3) and the shelf edge (RCP 4) were the most species‐rich, they may be characterised by a wider range of environmental responses, making them more challenging to define (Leaper et al. 2014). This transition zone between shallow and deep species is likely harder to predict due to the greater scale of responses present as noted by Hill et al. (2017) who found greater uncertainty associated with RCPs defined at intermediate depths (~400–700 m) of their study area (100–1200 m). Ecosystem types defined for the outer shelf of South Africa have been characterised by different sediment types (Sink, Harris, et al. 2019). Substrate type or percent sediment composition has been linked to global epifaunal distributions (Buhl‐Mortensen et al. 2012; Callaway et al. 2002; de Juan et al. 2013; Mayer and Piepenbung 1996; Murillo et al. 2016) and would likely improve finer scale delineation of bioregions across the outer shelf region here. Interestingly, the deep outer shelf bioregion also supported the highest number of indicator species, which may reflect the presence of a more heterogenous ‘mosaic’ habitat, as suggested by its partial alignment with the ‘Southern Benguela Mosaic Shelves’ (Sink, Harris, et al. 2019) and overlap with Childs Bank (de Wet and Compton 2021).

Another factor that may influence epifaunal patterns across bioregions is commercial trawling. Along the Southern Benguela shelf, areas subjected to heavy or frequent trawling have been shown to support altered epifaunal assemblages, with reduced richness and abundance compared to lightly trawled areas (Atkinson, Field, and Hutchings 2011). Because trawling pressure is not evenly distributed and may covary with depth, substrate, and other environmental features, some of the community differences observed here could partly reflect trawl impacts. Incorporating spatially explicit data on trawl intensity in future modelling would help to distinguish natural drivers from anthropogenic impacts. It is worth noting that including more variables will only improve model accuracy and predictions up to a point, whereafter the inclusion of additional variables does not provide much improvement and may further complicate the interpretation (Leathwick, Elith, Francis, et al. 2006).

The authors of previous local classifications have noted the limitations of the expert‐driven approach, specifically calling for the use of data‐driven approaches to iteratively improve ecosystem type maps in South Africa (Sink et al. 2023). Utilising a simultaneous approach to classification and prediction, the RCP framework applied here is a rigorous method with practical applications. Bioregional patterns are described based on their species profiles and environmental responses, which are put into greater context by estimated uncertainty maps. This method lends itself well when biological samples have been systematically collected across many sites. Patterns of species abundance are useful for management and conservation since maxima and minima of species abundance distributions can be identified, and highly abundant species can serve as potential indicators of bioregions.

Multi‐species models can estimate patterns of rare species more successfully than single‐species models (Hui et al. 2013; Leathwick, Elith, and Hastie 2006; Ovaskainen and Soininen 2011), though extremely rare species are generally excluded since they can add noise to the model without offering much additional information for modelling (Foster et al. 2017; Hui et al. 2013). Many species were removed from this analysis due to apparent rarity (76%), and it is unknown to what extent a reduction in the dataset could have been misleading. When only common species are used, bioregional patterns may appear more similar across groups that share a highly abundant species and less similar across groups that don't. However, simplification is essential for management (Carney 2005) and identifying indicator species is easier when common species are the focus (Murillo et al. 2016). Perhaps of greater interest is that apparent rarity was high in this region, which although common in marine environments, could be indicative of sampling inefficiency or patchiness (Williams et al. 2010). As such, a greater number of sites may be required to determine bioregions using this approach when apparent rarity is high.

Model diagnostics were performed to check whether model performance was reasonable, but without validation using independent datasets, evaluating how well bioregions approximated ‘real’ assemblages was difficult. Validation with independent data was beyond the scope of this study, having limited access to reliable epifaunal datasets. However, the temporal regularity of which demersal research trawls are conducted on the west (and south) continental shelf in this region provides good opportunities for validating models in the future.

## 5 Conclusion

RCP models defined five bioregions for the Southern Benguela margin on the South African west coast, based on epifaunal abundance collected by trawl surveys. Clear bathymetric breaks corresponded to the separation of the mid and outer shelves (~150 m) and to the shelf‐slope transition (400–500 m). RCP 1 (< ~150 m) and RCP 5 (> 400–500 m) were associated with the highest degrees of certainty in spatial predictions and occurred at sharp decreases in bottom oxygen concentration and temperature, respectively. Distinct communities were associated with these bioregions, suggesting strong environmental gradients play an important role in driving broad‐scale epifaunal community patterns at these depths on the Southern Benguela margin. Bioregions located on the outer shelf at intermediate depths of the study area (RCP 2, 3 and 4) were dominated by the same species ( *Sympagurus dimorphus* ) and associated with greater uncertainty in spatial predictions. This suggests that at broad scales, environmental thresholds may be less pronounced across the outer shelf, though additional covariates (e.g., percent sediment composition) may be needed to refine bioregions here. RCP models provide a good framework for marine bioregionalization, provided an adequate set of data is used. This may be even more relevant when apparent rarity is high (as in this study) to avoid potentially misleading results from only focusing on common species. Applying abundance data to inform bioregionalizations provides a greater depth of understanding into species distributions and can identify potential indicator species for bioregions. This study fills a knowledge gap in the application of trawled epifaunal abundance data for informing data‐driven marine bioregionalizations in South Africa. Maps produced and methods applied here can inform data‐driven frameworks for marine ecosystem classification and mapping in South Africa.

## Author Contributions

**Donia Wozniak:** data curation (lead), formal analysis (lead), funding acquisition (supporting), investigation (equal), methodology (equal), project administration (supporting), resources (supporting), software (lead), supervision (supporting), validation (equal), visualization (lead), writing – original draft (lead), writing – review and editing (equal). **Lara Atkinson:** conceptualization (equal), data curation (lead), formal analysis (supporting), funding acquisition (supporting), investigation (supporting), methodology (equal), project administration (equal), resources (equal), software (supporting), supervision (equal), validation (equal), visualization (supporting), writing – original draft (supporting), writing – review and editing (equal). **Natasha Karenyi:** conceptualization (equal), data curation (supporting), formal analysis (supporting), funding acquisition (lead), investigation (equal), methodology (equal), project administration (equal), resources (equal), software (supporting), supervision (equal), validation (equal), visualization (supporting), writing – original draft (supporting), writing – review and editing (equal).

## Funding

This work was supported by the National Research Foundation (116038 and 138572).

## Conflicts of Interest

The authors declare no conflicts of interest.

## Supporting information


**Data S1:** ece372549‐sup‐0001‐supinfo.docx.

## Data Availability

All data used in this study is available for review on South African Environment Observation Network Data Portal https://doi.org/10.15493/SAEON.EGAGASINI.20250312.
