# Supplementary material for: Statistically Classified Marine Epifaunal Bioregions on the Southern Benguela Margin, South Africa
Source: Ecol Evol. 2025 Dec 9;15(12):e72549. doi: 10.1002/ece3.72549 (PMC12690162; doi:10.1002/ece3.72549)
Supplement: Supplementary file 1 — Data S1: ece372549‐sup‐0001‐supinfo.docx. [file ECE3-15-e72549-s001.docx]

Supplementary Information

Statistically determined bioregions for the western continental margin of South Africa

Appendix S1. Species occurrences

**Table S1.1** List of the 46 species chosen for RCP modelling and their total abundances and occurrences across 325 sites.

| Species | Total Abundance | Occurrence | % Occurrence |
| --- | --- | --- | --- |
| *Actinauge granulata* | 456 | 80 | 24.62 |
| *Actinostola capensis* | 2034 | 114 | 35.08 |
| *Anthoptilum grandiflorum* | 296 | 24 | 7.38 |
| *Aphrodita alta* | 58 | 37 | 11.38 |
| *Astropecten irregularis pontoporeus* | 642 | 100 | 30.77 |
| *Athleta abyssicola* | 91 | 55 | 16.92 |
| *Athleta lutosa* | 123 | 32 | 9.85 |
| *Bolocera kerguelensis* | 409 | 49 | 15.08 |
| *Brissopsis lyrifera capensis* | 2319 | 113 | 34.77 |
| *Cavernularia* spp. | 9323 | 28 | 8.62 |
| *Chaceon chuni* | 2738 | 40 | 12.31 |
| *Cheiraster hirsutus* | 3447 | 87 | 26.77 |
| *Cosmasterias felipes* | 196 | 53 | 16.31 |
| *Crossaster penicillatus* | 27443 | 130 | 40.00 |
| *Diplopteraster multipes* | 325 | 72 | 22.15 |
| *Dipsacaster sladeni capensis* | 330 | 73 | 22.46 |
| *Dorhynchus thomsoni* | 659 | 87 | 26.77 |
| *Echinus gilchristi* | 132 | 41 | 12.62 |
| *Exodromidia spinosa* | 2594 | 142 | 43.69 |
| *Funchalia woodwardi* | 1129 | 36 | 11.08 |
| *Fusitriton magellanicus* | 478 | 146 | 44.92 |
| *Henricia abyssalis* | 77 | 47 | 14.46 |
| *Hyalinoecia tubicola* | 576 | 23 | 7.08 |
| *Luidia sarsii africana* | 696 | 147 | 45.23 |
| *Mediaster bairdi capensis* | 115 | 38 | 11.69 |
| *Mursia cristiata* | 409 | 107 | 32.92 |
| *Neptuneopsis gilchristi* | 38 | 20 | 6.15 |
| *Ophiomyxa vivipara capensis* | 91 | 23 | 7.08 |
| *Ophiothrix aristulata* | 275 | 43 | 13.23 |
| *Ophiura trimeni* | 503 | 27 | 8.31 |
| *Ophiuroglypha costata* | 527 | 61 | 18.77 |
| *Parapagurus bouvieri* | 4989 | 57 | 17.54 |
| *Pasiphaea* sp. 1 | 95649 | 31 | 9.54 |
| *Perissasterias polyacantha* | 64 | 34 | 10.46 |
| *Plesionika martia* | 2309 | 43 | 13.23 |
| *Pseudarchaster tessellatus* | 555 | 109 | 33.54 |
| *Psilaster acuminatus* | 1661 | 152 | 46.77 |
| *Pteraster capensis* | 170 | 40 | 12.31 |
| *Pterygosquilla capensis* | 22900 | 136 | 41.85 |
| *Scyramathia hertwigi* | 306 | 65 | 20.00 |
| *Sergia* spp. | 4393 | 24 | 7.38 |
| *Solenocera africana* | 111 | 26 | 8.00 |
| *Spatangus capensis* | 3391 | 97 | 29.85 |
| *Sympagurus dimorphus* | 107557 | 152 | 46.77 |
| *Toraster tuberculatus* | 444 | 84 | 25.85 |
| Velutinid | 261 | 59 | 18.15 |

Appendix S2. Environmental covariates


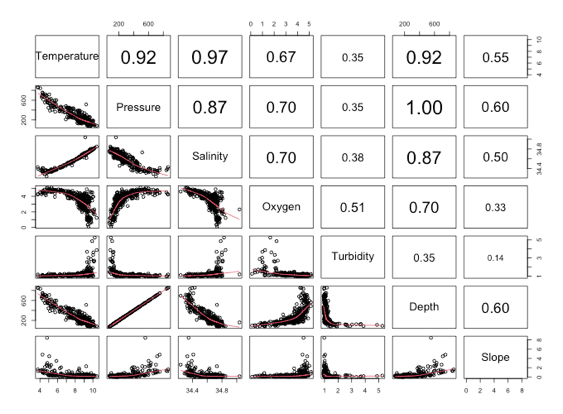
**Figure S2.1** Pairs plots and Pearson correlation coefficients (r) for environmental covariates considered for modelling.

**
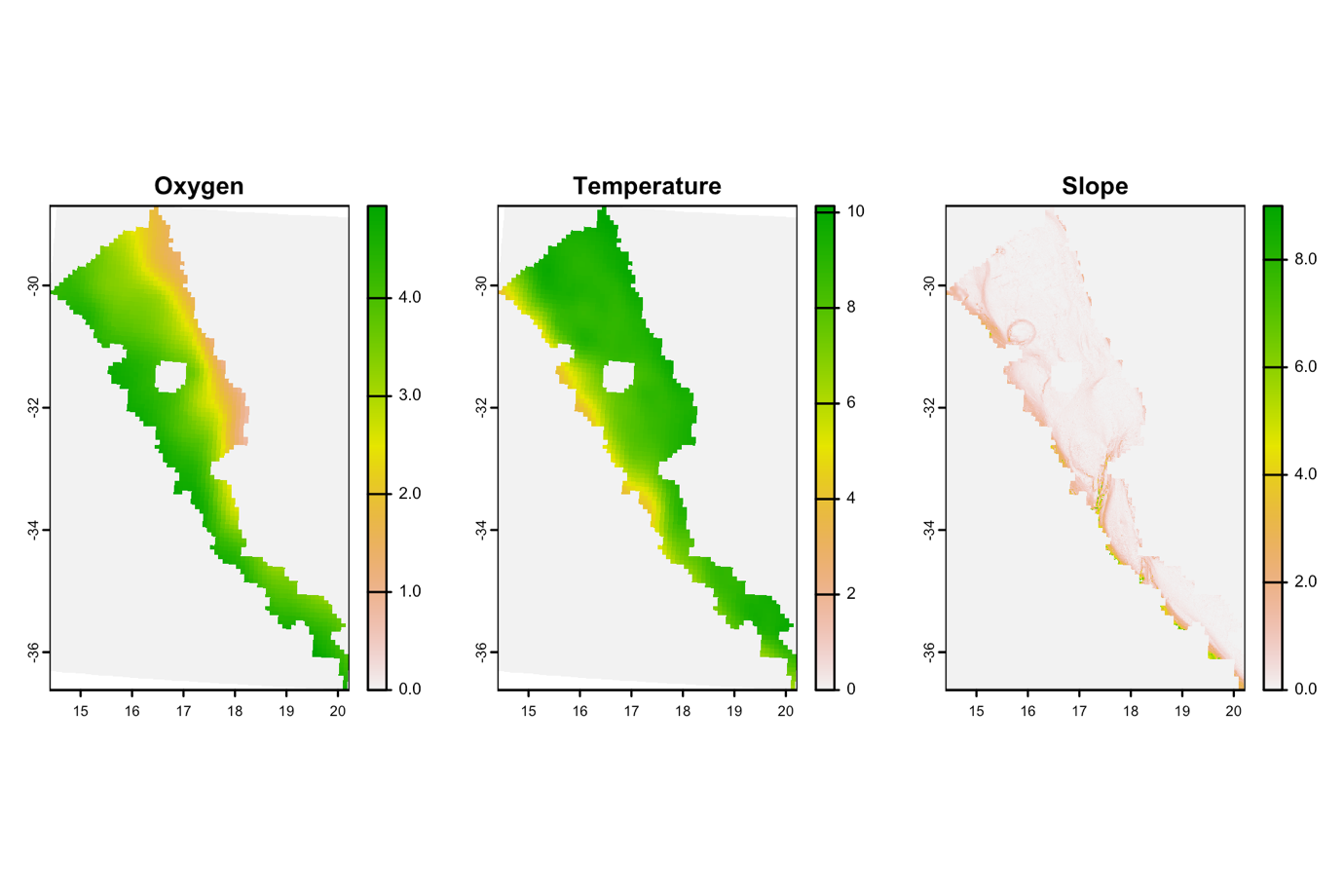
Figure S2.2.** Environmental space used for predicting RCPs. Bottom oxygen (ml/l) and bottom temperature (˚C) raster layers were based on *in-situ* CTD collected data from the same research trawl surveys as the biological data and kriged across the study area. The blank space in kriged layers represents an area where limited samples were available for kriging. Slope (˚) was derived from bathymetry ([de Wet & Compton, 2021](#_ENREF_51)) in ArcGIS Pro (ESRI).

Appendix S3. Model assessment and diagnostic plots

*Group selection*


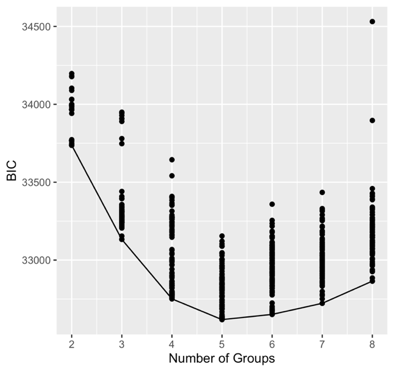
**Figure S3.3** BIC values for models with 2–8 RCP groups, run with 100 random starts, using temperature + oxygen + slope as environmental predictor variables. Values are based on a model using 46 epifaunal species from 325 sites. The best model minimizes BIC (5 groups).

*Random quantile residuals & QQ plots*

To check whether the model reasonably captured the variation in the data, random quantile residuals ([RQR, Dunn & Smyth, 1996](#_ENREF_57)) adapted for mixture models ([Dunstan et al., 2013](#_ENREF_59)) were inspected. The distribution of the RQR should be standard normal if the model performs well. To visually inspect homogeneity of variance in residuals, RQR were plotted against fitted values. Quantile-Quantile (QQ) plots were used to check for normality of residuals.

Random quantile residuals (RQR) were approximately normally distributed for the best model fitted and variation was reasonably well captured in the model **(Figure S3.4)**.


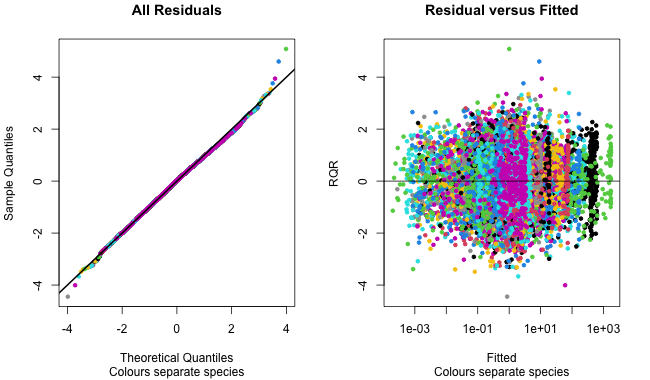
**Figure S3.4** Randomised quantile residual (RQR) diagnostic plots (calculated using Smyth-Dunn residuals). Left: quantile-quantile (QQ) plot testing for normality and Right: RQR against fitted values testing for homogeneity of variance in residuals. Colours are reused between the 46 species.

*Hold-out tests (Cook’s distance metric)*

Since RCP groups are latent (unobserved), traditional validation of how well the RCP groups represent the data is difficult to perform. However, the robustness and stability of RCP groups and log-likelihoods can be assessed by removing subsets of the data of increasing size. The Cook’s distance metric ([Cook, 1979](#_ENREF_41)) was used to assess the stability of RCP groups, as adapted by [Foster et al. (2017)](#_ENREF_77). Plots indicate how much predicted RCP membership probabilities (on average) change after a certain subset of data is removed, with larger values indicating a greater instability of RCP groups. The predictive log-likelihood was also plotted against hold-out sample size (i.e. number of sites removed) as an additional visual check of model stability. A notable drop or variability in the predictive log-likelihood as subsets of the data are removed indicates an unstable model, one that will be poor at predicting new observations ([Foster et al., 2017](#_ENREF_77)).

Hold-out tests (removing subsamples of sites) assessing the robustness of RCPs indicated an adequate performance, as indicated by the relatively small changes in the predictive log-likelihood (black line, **Figure S3.5 right**). The deepest (RCP 5, dark green line) and shallowest (RCP 1, light green line) bioregions were most stable with respect to removing subsets of the data **(Figure S3.5 left).** Although the predictive log-likelihood initially decreased, it remained relatively stable and did not fluctuate much, indicating acceptable stability with respect to removing subsets of the data **(Figure S3.5 right).**

**
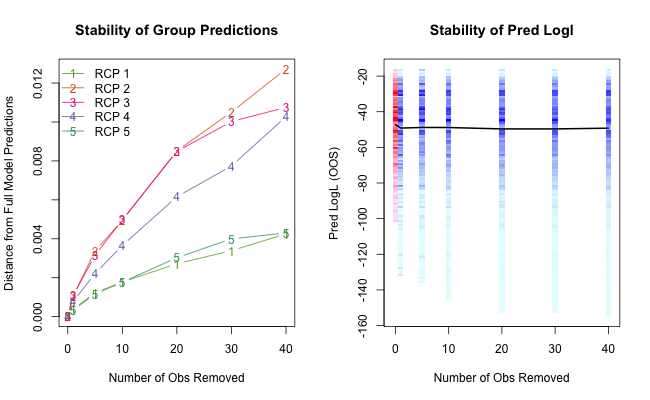
Figure S3.5** Diagnostic plots assessing stability of RCP groups, based on Cook’s distance (left) and predictive log-likelihood (right), against hold-out sample size. The predictive log-likelihood of the final model is indicated in red colours and with samples removed in blue colours.

Appendix S4. Estimated species abundances

**Table S4.2** Species catch profiles for each bioregion (RCP 1–5). Profiles based on mean predicted abundances (individuals per 30-minute trawl tow) ± lower and upper confidence intervals (CI).

|  | **RCP 1: Mid shelf** | | | **RCP 2: Shallow outer shelf** | | | **RCP 3: Deep outer shelf** | | | **RCP 4: Shelf edge** | | | **RCP 5: Upper slope** | | |
| --- | --- | --- | --- | --- | --- | --- | --- | --- | --- | --- | --- | --- | --- | --- | --- |
| Species | Lower CI | Mean | Upper CI | Lower CI | Mean | Upper CI | Lower CI | Mean | Upper CI | Lower CI | Mean | Upper CI | Lower CI | Mean | Upper CI |
| *Actinauge granulata* | 0.0008 | 0.001 | 0.0013 | 0.0315 | 0.087 | 0.1777 | 0.1317 | 0.4104 | 0.7953 | 3.0742 | 5.0397 | 7.7647 | 0.9288 | 2.336 | 4.6827 |
| *Actinostola capensis* | 0.0014 | 0.0362 | 0.0965 | 0.0008 | 0.0829 | 0.3087 | 0.7861 | 1.7038 | 2.5729 | 15.156 | 29.4765 | 57.5661 | 1.8614 | 5.1367 | 15.9404 |
| *Anthoptilum grandiflorum* | 0.0004 | 0.0006 | 0.0008 | 0.0001 | 0.0045 | 0.0326 | 0.5747 | 3.0789 | 9.4921 | 0.0244 | 1.2747 | 5.5908 | 0.0002 | 0.0006 | 0.0011 |
| *Aphrodita alta* | 0.0007 | 0.0008 | 0.001 | 0.0339 | 0.0867 | 0.1574 | 0.1937 | 0.4549 | 0.8263 | 0.0463 | 0.197 | 0.4125 | 0.0243 | 0.1288 | 0.2814 |
| *Astropecten irregularis pontoporeus* | 2.5019 | 4.1753 | 6.3886 | 1.8195 | 4.0757 | 9.2476 | 0.2465 | 0.7524 | 1.8441 | 0.0007 | 0.0014 | 0.0037 | 0.0019 | 0.0343 | 0.1056 |
| *Athleta abyssicola* | 0.0035 | 0.0749 | 0.2333 | 0.1682 | 0.2928 | 0.5302 | 0.0736 | 0.2558 | 0.6153 | 0.4905 | 0.7706 | 1.1156 | 0.0006 | 0.0008 | 0.001 |
| *Athleta lutosa* | 1.1471 | 2.0646 | 3.3681 | 0.0128 | 0.116 | 0.3718 | 0.0004 | 0.032 | 0.122 | 0.0004 | 0.0011 | 0.006 | 0.0004 | 0.0006 | 0.0008 |
| *Bolocera kerguelensis* | 0.0024 | 0.0491 | 0.1722 | 0.0019 | 0.4065 | 0.9636 | 1.6034 | 4.1964 | 8.1498 | 0.5032 | 1.3216 | 2.5795 | 0.0876 | 0.2105 | 0.3982 |
| *Brissopsis lyrifera capensis* | 0.2541 | 2.184 | 4.4291 | 2.6344 | 14.7564 | 43.8552 | 1.7301 | 5.5096 | 17.4912 | 1.06 | 11.437 | 34.3402 | 0.0044 | 0.0518 | 0.1567 |
| *Cavernularia* spp. | 70.7034 | 183.8137 | 384.048 | 0.0002 | 0.0133 | 0.0684 | 0.0003 | 0.0008 | 0.0034 | 0.0004 | 0.0006 | 0.0009 | 0.0004 | 0.0005 | 0.0007 |
| *Chaceon chuni* | 0.0005 | 0.0006 | 0.0008 | 0 | 0.0217 | 0.1228 | 0.0003 | 0.0005 | 0.0008 | 0.001 | 0.0381 | 0.1601 | 31.1033 | 48.3005 | 72.7846 |
| *Cheiraster hirsutus* | 0.0009 | 0.0011 | 0.0014 | 0.0435 | 0.276 | 0.667 | 0.5613 | 1.5057 | 2.9625 | 21.2473 | 61.6623 | 136.8838 | 0.1273 | 0.6451 | 1.6018 |
| *Cosmasterias felipes* | 0.0007 | 0.0009 | 0.0011 | 0.0747 | 0.4078 | 0.813 | 0.8489 | 1.7959 | 3.3475 | 0.1301 | 0.6506 | 1.4241 | 0.0047 | 0.0356 | 0.0812 |
| *Crossaster penicillatus* | 0.0011 | 0.0033 | 0.0353 | 0.2413 | 0.9401 | 3.0243 | 0.7491 | 3.2244 | 6.0502 | 31.9969 | 83.8127 | 168.3416 | 246.4622 | 413.2448 | 667.0117 |
| *Diplopteraster multipes* | 0.0007 | 0.001 | 0.0013 | 0.0003 | 0.0858 | 0.2094 | 0.2664 | 1.0469 | 1.8748 | 1.7074 | 2.7741 | 4.3448 | 0.7074 | 1.6336 | 2.9151 |
| *Dipsacaster sladeni capensis* | 0.0008 | 0.001 | 0.0012 | 0.0143 | 0.1448 | 0.4053 | 0.4111 | 1.0402 | 2.0812 | 2.5985 | 4.2514 | 6.0545 | 0.0185 | 0.1729 | 0.6069 |
| *Dorhynchus thomsoni* | 0.0126 | 0.0918 | 0.2202 | 0.048 | 0.2138 | 0.3838 | 0.9479 | 2.766 | 5.2853 | 2.7611 | 5.8055 | 11.1603 | 0.6597 | 2.2985 | 5.1567 |
| *Echinus gilchristi* | 0.0007 | 0.0134 | 0.158 | 0.0141 | 0.2486 | 0.6977 | 0.591 | 1.0614 | 1.8742 | 0.0321 | 0.586 | 1.5063 | 0.0018 | 0.0343 | 0.1231 |
| *Exodromidia spinosa* | 7.9057 | 14.5247 | 23.5145 | 2.5378 | 4.6865 | 12.3036 | 0.7286 | 1.4153 | 2.3836 | 1.8164 | 25.9432 | 79.0344 | 0.0012 | 0.0177 | 0.0693 |
| *Funchalia woodwardi* | 0.0005 | 0.0006 | 0.0008 | 0.0003 | 0.0004 | 0.0006 | 0.0004 | 0.0005 | 0.0007 | 0.0184 | 1.4652 | 6.4786 | 10.5844 | 18.1284 | 27.9594 |
| *Fusitriton magellanicus* | 0.8837 | 1.6131 | 2.9387 | 0.707 | 1.2051 | 1.9668 | 0.7773 | 1.5892 | 2.4503 | 1.2609 | 1.9583 | 2.7255 | 0.5154 | 1.1673 | 1.907 |
| *Henricia abyssalis* | 0.0027 | 0.0392 | 0.111 | 0.0678 | 0.1823 | 0.3327 | 0.1093 | 0.3769 | 0.6743 | 0.3473 | 0.6158 | 0.9482 | 0.0005 | 0.0024 | 0.0173 |
| *Hyalinoecia tubicola* | 0.0003 | 0.0004 | 0.0004 | 0.0002 | 0.0003 | 0.0004 | 0.0002 | 0.0003 | 0.0004 | 0.0003 | 0.0004 | 0.0005 | 5.6984 | 10.0372 | 16.3247 |
| *Luidia sarsii africana* | 0.0937 | 0.2207 | 0.4207 | 1.0753 | 1.6348 | 2.3183 | 1.9582 | 3.0675 | 4.4954 | 3.4932 | 5.9399 | 9.821 | 0.0543 | 0.1742 | 0.3277 |
| *Mediaster bairdi capensis* | 0.0005 | 0.0144 | 0.0822 | 0.6336 | 1.0651 | 1.6281 | 0.0065 | 0.0783 | 0.4528 | 0.0142 | 0.1566 | 0.5274 | 0.0005 | 0.0352 | 0.138 |
| *Mursia cristiata* | 0.1427 | 0.4481 | 1.2162 | 0.5502 | 0.9556 | 2.6845 | 0.4582 | 1.0973 | 1.7419 | 1.0223 | 3.722 | 9.5424 | 0.0119 | 0.0644 | 0.1837 |
| *Neptuneopsis gilchristi* | 0.0003 | 0.0005 | 0.0006 | 0.0002 | 0.0934 | 0.3223 | 0.1999 | 0.4378 | 0.8023 | 0.0003 | 0.001 | 0.0073 | 0.0002 | 0.0005 | 0.0007 |
| *Ophiomyxa vivipara capensis* | 0.0004 | 0.0005 | 0.0007 | 0.0002 | 0.0006 | 0.0009 | 0.3163 | 0.9875 | 1.7514 | 0.0228 | 0.4071 | 1.0286 | 0.0003 | 0.0005 | 0.0007 |
| *Ophiothrix aristulata* | 0.0074 | 0.0498 | 0.1185 | 0.0215 | 0.1405 | 0.358 | 1.712 | 3.6454 | 5.7733 | 0.0006 | 0.091 | 0.5095 | 0.0005 | 0.0007 | 0.0009 |
| *Ophiura trimeni* | 0.0007 | 0.0009 | 0.0011 | 0.0004 | 0.0006 | 0.0008 | 0.0072 | 2.0935 | 8.0868 | 1.1677 | 4.2115 | 9.4038 | 0.8242 | 2.3277 | 4.2826 |
| *Ophiuroglypha costata* | 0.0006 | 0.0009 | 0.0013 | 0.1657 | 0.2837 | 0.4552 | 2.8436 | 6.2734 | 12.5328 | 0.419 | 1.0148 | 1.827 | 0.0006 | 0.0008 | 0.001 |
| *Parapagurus bouvieri* | 0.0008 | 0.001 | 0.0013 | 0.0005 | 0.0007 | 0.001 | 0.0278 | 6.1807 | 14.5438 | 26.1055 | 79.0278 | 158.2851 | 1.472 | 4.3314 | 8.4358 |
| *Pasiphaea* sp. 1 | 829.6363 | 1906.2752 | 3377.2839 | 0.0004 | 0.0007 | 0.0017 | 0.0007 | 0.0136 | 0.047 | 0.0006 | 0.0009 | 0.0016 | 0.2005 | 0.6806 | 1.5518 |
| *Perissasterias polyacantha* | 0.0029 | 0.0323 | 0.106 | 0.0825 | 0.1938 | 0.3113 | 0.3541 | 0.598 | 0.963 | 0.0016 | 0.0501 | 0.1382 | 0.0007 | 0.0176 | 0.0544 |
| *Plesionika martia* | 0.0007 | 0.0014 | 0.0101 | 0.0416 | 0.1878 | 0.5013 | 0.0005 | 0.0007 | 0.0009 | 0.0303 | 0.317 | 1.2018 | 20.0917 | 43.8777 | 90.329 |
| *Pseudarchaster tessellatus* | 0.373 | 1.4898 | 3.0276 | 2.2292 | 3.6115 | 5.5322 | 0.1453 | 0.3758 | 0.7473 | 0.8893 | 1.9218 | 3.5165 | 0.1397 | 0.3161 | 0.5747 |
| *Psilaster acuminatus* | 0.0017 | 0.1218 | 0.3577 | 0.3118 | 1.0616 | 1.6173 | 10.8841 | 18.3722 | 31.6668 | 2.1747 | 3.6133 | 5.6777 | 0.6953 | 1.3119 | 2.0699 |
| *Pteraster capensis* | 0.0007 | 0.0164 | 0.0749 | 0.1152 | 0.7314 | 1.7967 | 0.3036 | 1.1883 | 4.1454 | 0.1354 | 0.3363 | 0.6981 | 0.0131 | 0.0932 | 0.2152 |
| *Pterygosquilla capensis* | 176.9829 | 246.2346 | 343.8047 | 27.0843 | 110.6239 | 347.3573 | 0.7927 | 1.5985 | 2.9902 | 0.4552 | 2.0422 | 4.3466 | 0.0011 | 0.0031 | 0.0181 |
| *Scyramathia hertwigi* | 0.0008 | 0.001 | 0.0012 | 0.0278 | 0.09 | 0.1902 | 0.0633 | 0.5524 | 1.329 | 1.506 | 2.9157 | 5.3076 | 0.5917 | 1.7484 | 3.3813 |
| *Sergia* spp. | 0.0004 | 0.0004 | 0.0005 | 0.0002 | 0.0003 | 0.0005 | 0.0003 | 0.0004 | 0.0005 | 0.0003 | 0.0011 | 0.0065 | 43.1463 | 81.1325 | 129.5633 |
| *Solenocera africana* | 0.1604 | 0.5688 | 1.4727 | 0.4407 | 0.9158 | 1.721 | 0.0004 | 0.0229 | 0.0658 | 0.0029 | 0.0505 | 0.1464 | 0.0005 | 0.0008 | 0.001 |
| *Spatangus capensis* | 0.0011 | 0.0365 | 0.1187 | 1.5113 | 14.7677 | 27.6727 | 15.5423 | 27.4746 | 44.0561 | 0.3636 | 1.5689 | 3.274 | 0.0008 | 0.0011 | 0.0014 |
| *Sympagurus dimorphus* | 0.0153 | 0.1491 | 0.716 | 48.7552 | 363.2749 | 683.2187 | 153.6235 | 466.3162 | 893.7509 | 232.445 | 695.5396 | 1186.9874 | 0.2473 | 0.9799 | 2.0289 |
| *Toraster tuberculatus* | 0.0008 | 0.001 | 0.0012 | 1.6929 | 3.2715 | 4.8773 | 0.8089 | 1.5143 | 2.5842 | 0.3047 | 0.723 | 1.7342 | 0.0095 | 0.0683 | 0.1688 |
| Velutinid spp. | 0.0167 | 0.0597 | 0.1493 | 0.1869 | 0.619 | 1.3702 | 1.2581 | 2.4793 | 3.9117 | 0.177 | 0.5518 | 1.0984 | 0.0007 | 0.0009 | 0.0012 |


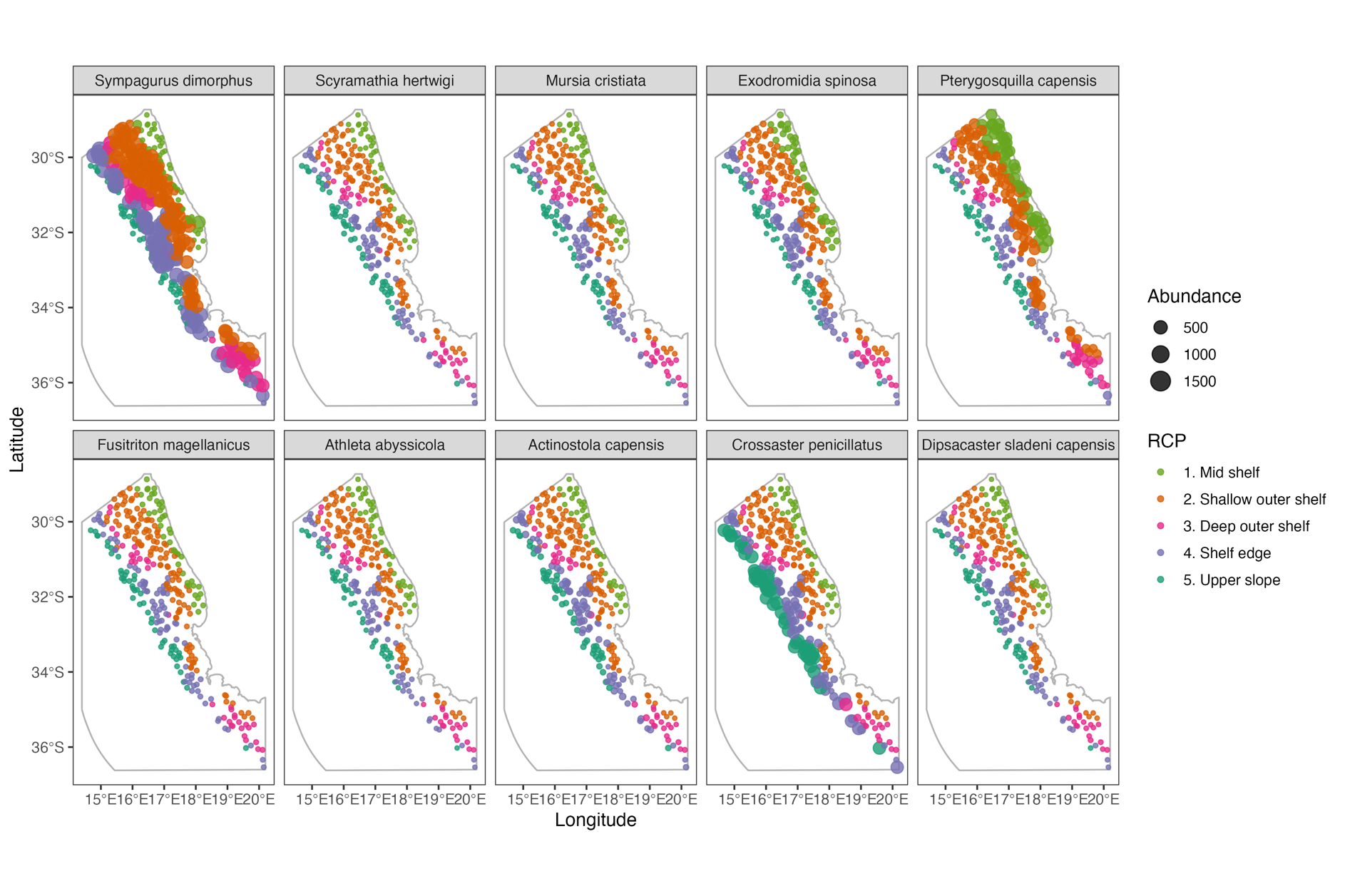
Figure S4.6 Estimated abundance per site of the 46 epifaunal species used for modelling, predicted for each RCP. Predicted abundance per site was calculated from model outputs assigned to bioregions according to the hard RCP classification and calculated for each species. Circle size represents abundance weight, with larger circles denoting a relatively greater abundance at that size.


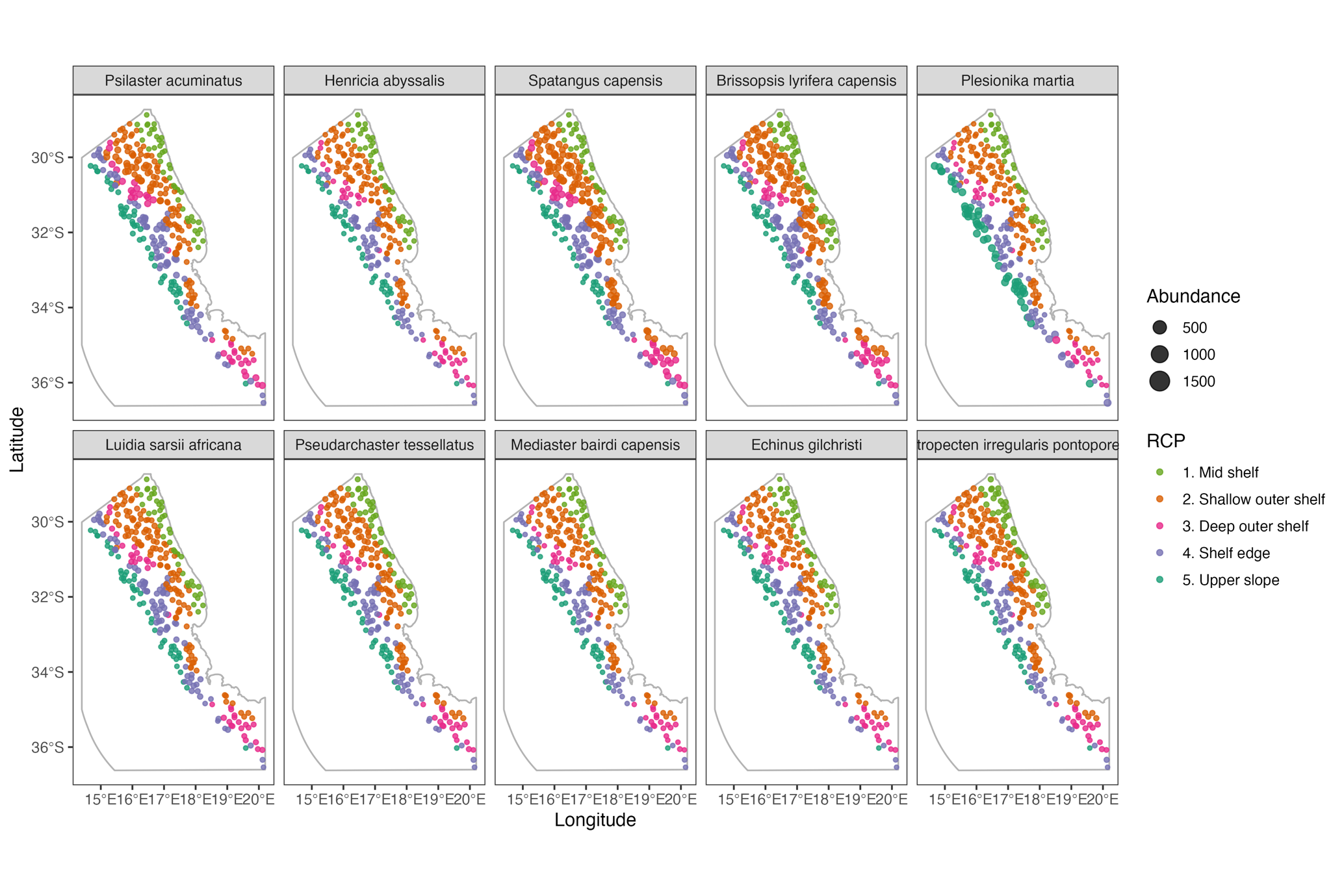
Figure S4.6 continued.


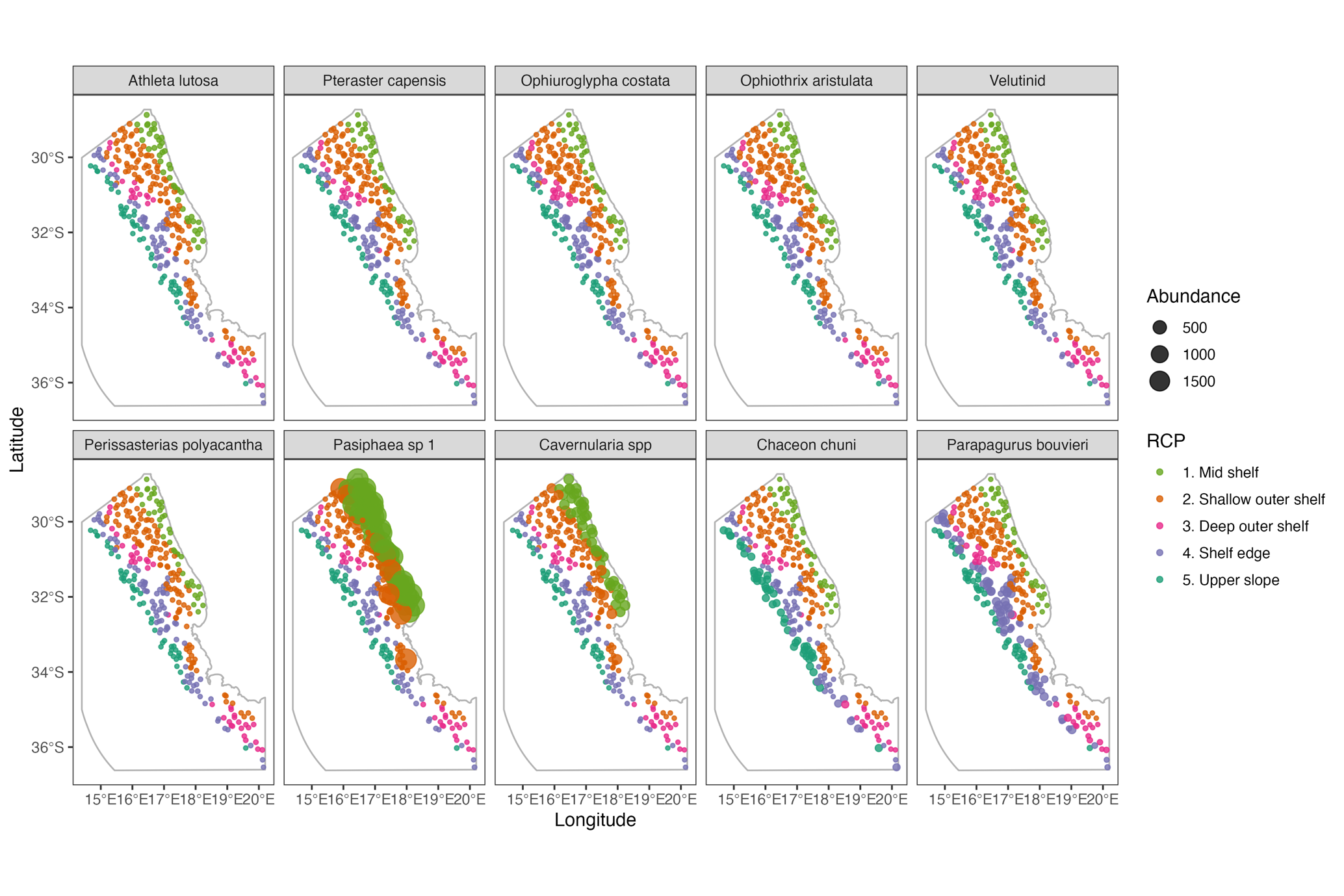
Figure S4.6 continued.


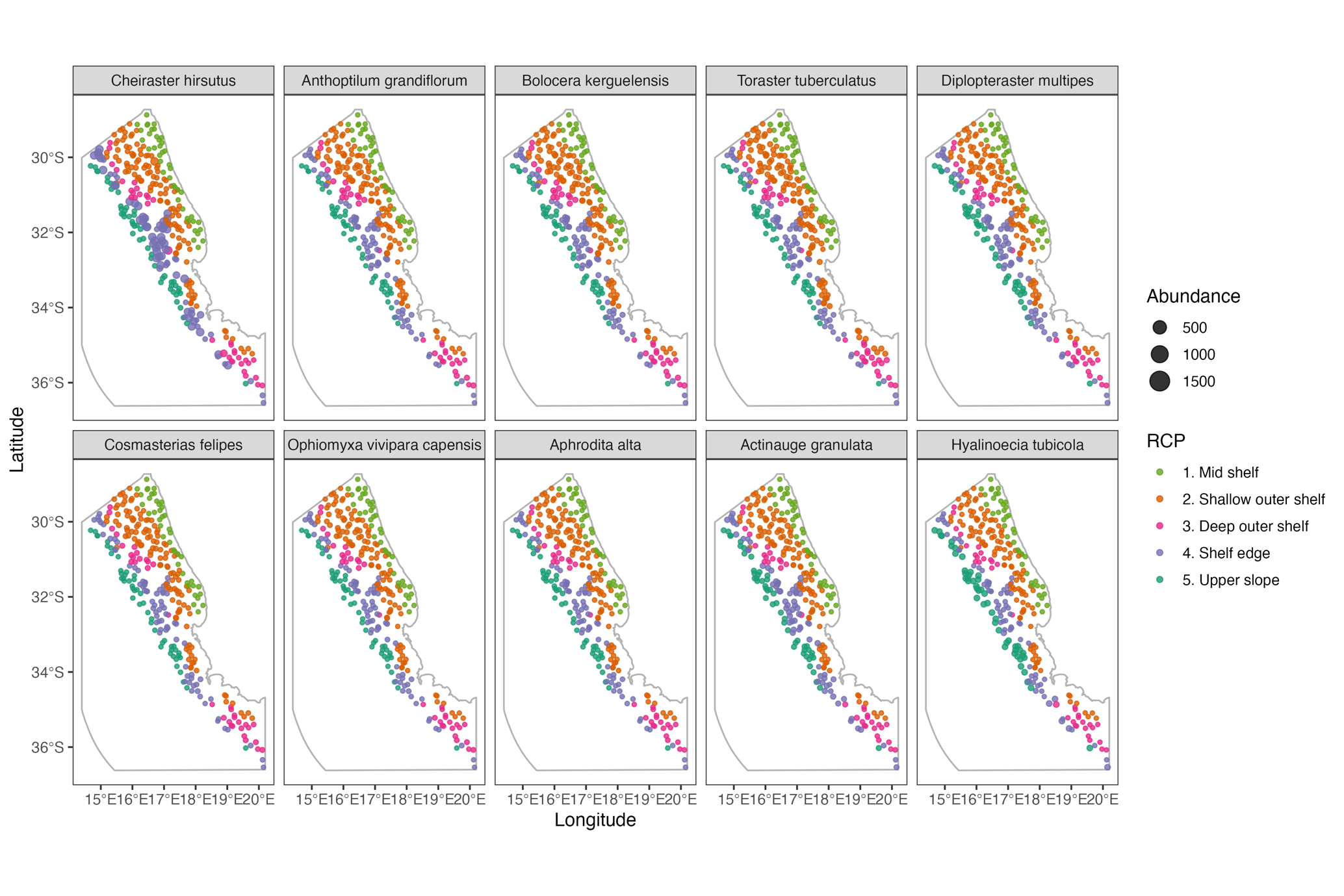
Figure S4.6 continued.


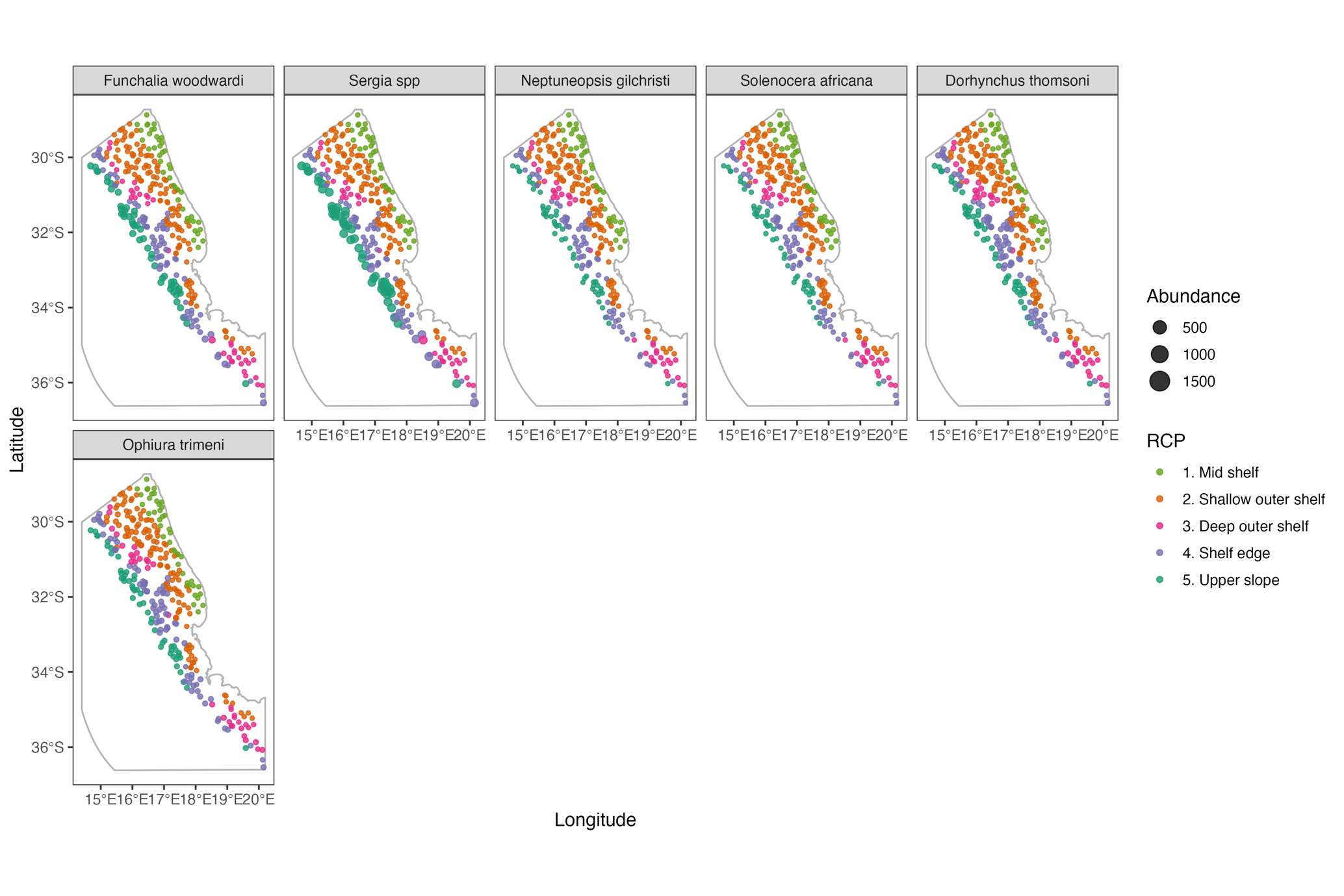
Figure S4.6 continued.
